# A crucial active site network of titratable residues guides catalysis and NAD ^+^ binding in human succinic semialdehyde dehydrogenase

**DOI:** 10.1002/pro.70024

**Published:** 2024-12-28

**Authors:** Samuele Cesaro, Marco Orlando, Ilaria Bettin, Carmen Longo, Giulia Spagnoli, Patrizia Polverino de Laureto, Gianluca Molla, Mariarita Bertoldi

**Affiliations:** ^1^ Department of Neuroscience, Biomedicine and Movement Sciences, Section of Biochemistry University of Verona Verona Italy; ^2^ Department of Biotechnology and Life Sciences University of Insubria Varese Italy; ^3^ Department of Biotechnology e Biosciences University of Milano‐Bicocca Milan Italy; ^4^ Department of Biology Friedrich‐Alexander University Erlangen‐Nürnberg Germany; ^5^ Department of Pharmaceutical and Pharmacological Sciences University of Padova Padova Italy

**Keywords:** bioinformatic and evolutionary analysis, enzymatic mechanism, kinetics, succinic semialdehyde dehydrogenase, succinic semialdehyde dehydrogenase deficiency

## Abstract

Human succinic semialdehyde dehydrogenase is a mitochondrial enzyme fundamental in the neurotransmitter γ‐aminobutyric acid catabolism. It catalyzes the NAD^+^‐dependent oxidative degradation of its derivative, succinic semialdehyde, to succinic acid. Mutations in its gene lead to an inherited neurometabolic rare disease, succinic semialdehyde dehydrogenase deficiency, characterized by mental and developmental delay. Due to the poor characterization of this enzyme, we carried out evolutionary and kinetic investigations to contribute to its functional behavior, a prerequisite to interpreting pathogenic variants. An in silico analysis shows that succinic semialdehyde dehydrogenases belong to two families, one human‐like and the other of bacterial origin, differing in the oligomeric state and in a network of active site residues. This information is coupled to the biophysical–biochemical characterization of the human recombinant enzyme uncovering that (i) catalysis proceeds by an ordered bi–bi mechanism with NAD^+^ binding before the aldehyde that exerts a partial non‐competitive inhibition; (ii) a stabilizing complex between the catalytic Cys340 and NAD^+^ is observed and interpreted as a protective mechanism; and (iii) a concerted non‐covalent network assists the action of the catalytic residues Cys340 and Glu306. Through mutational analyses of Lys214, Glu306, Cys340, and Glu515 associated with pH studies, we showed that NAD^+^ binding is controlled by the dyad Lys214‐Glu515. Moreover, catalysis is assured by proton transfer exerted by the same dyad networked with the catalytic Glu306, involved in catalytic Cys340 deprotonation/reprotonation. The identification of this weak bond network essential for cofactor binding and catalysis represents a first step to tackling the molecular basis for its deficiency.

## 1 INTRODUCTION

**SCHEME 1:**
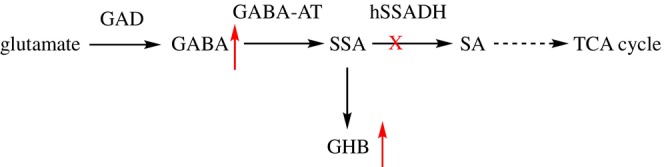
Metabolic pathways of GABA. The red cross denotes the misfunctioning or absence of hSSADH, leading to GABA and GHB accumulation (red arrows).

Human succinic semialdehyde dehydrogenase (hSSADH) (EC 1.2.1.24) is a mitochondrial enzyme belonging to the aldehyde dehydrogenase (ALDH) superfamily (Jackson et al. 2011). It plays a crucial role in the metabolism of γ‐aminobutyric acid (GABA) (Scheme 1). GABA is synthesized by decarboxylation of glutamate catalyzed by glutamate decarboxylase (GAD) and is then transaminated to succinic semialdehyde (SSA) by GABA aminotransferase (GABA‐AT). Thereafter, hSSADH catalyzes the oxidation of SSA into succinic acid (SA), with the concomitant reduction of oxidized nicotinamide adenine dinucleotide (NAD^+^) to its reduced form NADH. SA connects the GABA neurotransmitter pathway to the tricarboxylic acids (TCA) cycle, implying that the control of its level plays a distinct role in metabolism.

An impairment of hSSADH leads to increased amounts of both GABA and the catabolite γ‐hydroxybutyric acid (GHB), a toxic compound at high concentrations (Malaspina et al. 2009). About 70 variants, mainly missense, in *ALDH5A1*, the human gene coding for hSSADH (GRCh38: chr6:24,494,867–24,537,207; cytogenetic band 6p22.3), are responsible for a rare recessive genetic disorder, SSADH deficiency (OMIM #271980) identified in about 450 patients (Akaboshi et al. 2003; Brennenstuhl et al. 2020; DiBacco et al. 2020; Didiasova et al. 2024; Menduti et al. 2018; Pop et al. 2020). The symptoms include mental and developmental delay, ataxia, and hypotonia coupled with seizure, psychiatric episodes, and autism, which tend to worsen with age progression (Didiášová et al. 2020; Tokatly Latzer et al. 2023a; Tokatly Latzer et al. 2023b; Tokatly Latzer et al. 2024b). The pharmacological treatments are palliative and aimed to ameliorate symptoms (Tokatly Latzer et al. 2024a). A recent genotype‐to‐phenotype correlation based on structural analysis has been proposed (Tokatly Latzer et al. 2023c).

As with many other monogenic diseases, the broad spectrum of symptoms can also be due to the difference in variants that can affect the functionality of hSSADH by distinctive molecular mechanisms. Attempts to model the disease in mice by both enzyme replacement therapy of the wild‐type (WT) hSSADH or gene therapy with its cDNA (Lee et al. 2021; Lee et al. 2024) are ongoing. However, the knowledge of the structural determinants responsible for the activity of hSSADH is essential to develop an effective treatment.

**SCHEME 2:**
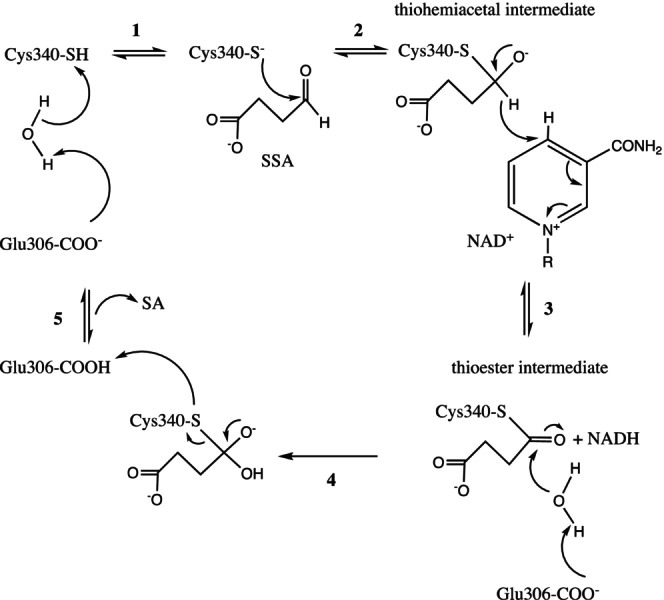
Proposed mechanism of reaction of hSSADH highlighting the role of Cys340 as a nucleophile and of Glu306 as acid–base catalyst. The catalytic Cys340 is deprotonated by the catalytic Glu306 (through a water molecule) (step 1) to generate a thiolate anion that (step 2) makes a nucleophilic attack to the formyl carbon of SSA. The produced thiohemiacetal intermediate transfers the hydride to NAD^+^, which is reduced to NADH (step 3) with the concomitant formation of the thioester intermediate. Glu306 acts as a general base (through a water molecule) to trigger the nucleophilic attack of a hydroxyl ion on the carbonyl carbon of the ester (step 4). This generates a tetrahedral intermediate with subsequent release of succinic acid (SA) and of Cys340 thiolate, which is finally reprotonated by Glu306 (step 5).

Surprisingly, hSSADH is a rather neglected enzyme. In literature, only a few papers (up to 1992) reported the investigation of the reaction carried out by the enzyme isolated from the human brain (Chambliss and Gibson 1992; Embree and Albers 1964; Ryzlak and Pietruszko 1988). More recently, the enzyme was cloned and purified in recombinant form (Chambliss et al. 1995; Kang et al. 2005) and obtained in high yields with a *k*_cat_ of about 1.7 s^−1^ and *K*_*m*_ for SSA and NAD^+^ of 6.3 and 125 μM, respectively (Kang et al. 2005). In the same paper, it was demonstrated that the enzyme was highly expressed in the brain, liver, skeletal muscle, and kidney. In 2009, the structure of hSSADH was solved (Kim et al. 2009) as a homotetramer (dimer of dimers) composed of four identical monomers, each of 535 residues. The monomeric subunit is composed of a mitochondrial peptide sequence (residues 1–47), a NAD^+^ binding domain (residues 48–173, 196–307, and 509–524), a catalytic (or substrate) domain (residues 308–508, containing the catalytic residues Cys340 and Glu306), and an oligomerization domain (residues 174–195 and 525–535). Although a reaction mechanism for hSSADH has not been determined yet, the generally accepted mechanism of ALDH (Kim et al. 2009; Phonbuppha et al. 2018) involves the essential catalytic roles of a cysteine and a glutamate residue (Cys340 and Glu306 for hSSADH) (Scheme 2).

The activity of hSSADH is further shown to be regulated by the redox state. Oxidizing conditions lead to inactivation with the formation of a disulfide bond between Cys340 and the adjacent Cys342 (Kim et al. 2009). This mechanism is reversible and would enable the sensitivity of the human enzyme to fluctuating redox conditions within the mitochondria (Kim et al. 2009). Only another SSADH of bacterial source has been recently reported to bear two nearby cysteine residues capable of a redox mechanism similar to the one proposed for the human enzyme (Paladkong et al. 2022; Phonbuppha et al. 2018).

Several homologous enzymes with SSA oxidation activity have been reported and biochemically characterized. Only a few of them are eukaryotic and show diversity in oligomerization state (dimer or tetramer), cofactor preference (NAD^+^ or NADP^+^) and kinetics (Table 1 and references therein), but a systematic evolutionary overview is lacking, considering also that the Aldehyde Dehydrogenase Gene Superfamily Resource Center is now inaccessible (Black and Vasiliou 2009).

**TABLE 1:** ALDH proteins coded by human *ALDH* genes and SSADH proteins from species other than *Homo sapiens* and for which a biochemical record is available.

Gene name	Protein name	ALDH family^a^	Source	GenBank protein ID	Length	Structure source^b^	Cofactor preference	Oligomerization state	Reference
*ALDH1A1*	RALDH 1	ALDH1	*Homo sapiens*	AAA51692	501	PDB: 4WJ9	NAD^+^(H)	Tetramer	Koch et al. (2015)
*ALDH1A2*	RALDH 2	ALDH1	*Homo sapiens*	BAA34785	518	PDB: 4X2Q	NAD^+^(H)	Tetramer	Zhao et al. (1996)
*ALDH1A3*	RALDH‐3	ALDH1	*Homo sapiens*	AAA79036	512	PDB: 5FHZ	NAD^+^(H)	Tetramer	Moretti et al. (2016)
*ALDH1B1*	ALDH1B1	ALDH1	*Homo sapiens*	AAA96830	517	AFDB: AF‐P30837‐F1	NAD^+^(H)	NA	Stagos et al. (2010)
*ALDH1L1*	FDH	ALDH1L	*Homo sapiens*	AAC35000	902	AFDB: AF‐Q3SY69‐F1	NADP^+^(H)	NA	NA
*ALDH1L2*	mtFDH	ALDH1L	*Homo sapiens*	AAI03935	923	AFDB: AF‐Q3SY69‐F1	NADP^+^(H)	NA	Strickland et al. (2011)
*ALDH2*	ALDH‐E2	ALDH2	*Homo sapiens*	AAA51693	517	PDB: 1O05	NAD^+^(H)	Tetramer	Hurley et al. (2001)
*ALDH3A1*	ALDHIII	ALDH3	*Homo sapiens*	AAA51696	453	PDB: 3SZA	NAD^+^(H)	Dimer	Hsu et al. (1992)
*ALDH3A2*	FALDH	ALDH3	*Homo sapiens*	AAB01003	485	PDB: 4QGK	NAD^+^(H)	Dimer	Kelson et al. (1997)
*ALDH3B1*	ALDH3B1	ALDH3	*Homo sapiens*	AAA83428	468	AFDB: AF‐P43353‐F1	NADP^+^(H)	NA	Kitamura et al. (2013) and Marchitti et al. (2007)
*ALDH3B2*	ALDH3B2	ALDH3	*Homo sapiens*	AAA85441	385	AFDB: AF‐P48448‐F1	NAD^+^(H)	NA	NA
*ALDH4A1*	P5CDH	ALDH4	*Homo sapiens*	AAC50501	563	PDB: 3V9G	NAD^+^(H)	Dimer	Srivastava et al. (2012)
*ALDH5A1*	*Hs*SSADH	ALDH5	*Homo sapiens*	CAA72076	535	PDB: 2W8O	NAD^+^(H)	Tetramer	Kim et al. (2009), this study
*ALDH6A1*	MMSDH	ALDH6	*Homo sapiens*	CAB76468	535	AFDB: AF‐Q02252‐F1	NAD^+^(H)	NA	NA
*ALDH7A1*	Alpha‐AASA	ALDH7	*Homo sapiens*	NP_001173	539	PDB: 4ZVX	NADP^+^(H)	Tetramer	Brocker et al. (2010)
*ALDH8A1*	ALDH8A1	ALDH8	*Homo sapiens*	AAG42417	487	AFDB: AF‐Q9H2A2‐F1	NAD^+^(H)	NA	Hsu et al. (1999)
*ALDH9A1*	ALDH9A1	ALDH9	*Homo sapiens*	AAF43600	494	PDB: 6QAP	NAD^+^(H)	Tetramer	Kikonyogo and Pietruszko (1996) and Koncitikova et al. (2019)
*ALDH16A1*	ALDH16A1	ALDH16	*Homo sapiens*	AAH14895	802	AFDB: AF‐Q8IZ83‐F1	NA	NA	NA
*ALDH18A1*	P5CS	ALDH18	*Homo sapiens*	CAA64224	795	PDB: 2H5G	AD(T)P	Dimer	Hu et al. (1999)
gabD1	mtGabD1	Other SSADHs	*Mycobacterium tuberculosis*	WP_003912534	457	AFDB: AF‐P9WNX9‐F1	NADP^+^(H)	NA	de Carvalho et al. (2011)
gabD	SySSADH	Other SSADHs	*Synechocystis* 6803	BAA10090	454	AFDB: AF‐Q55585‐F1	NADP^+^(H)	NA	Ito and Osanai (2020)
gabD	SpSSADH	Other SSADHs	*Streptococcus pyogenes*	WP_014407552	465	PDB: 4OGD	NADP^+^(H)	Dimer	Jang et al. (2014)
gabD	BsSSADH	ALDH5 (Human‐like SSADHs)	*Bacillus subtilis*	NP_388273	462	AFDB: AF‐P94428‐F1	NADP^+^(H)/ NAD^+^(H)	NA	Park et al. (2014)
gabD	DmSSADH	ALDH5 (Human‐like SSADHs)	*Drosophila melanogaster*	NP_651408	509	AFDB: AF‐Q9VBP6‐F1	NAD^+^(H)	NA	Rothacker and Ilg (2008)
gabD	AbSSADH	ALDH5 (Human‐like SSADHs)	*Acinetobacter baumanii*	ANA38811	482	AF	NADP^+^(H)	Tetramer	Phonbuppha et al. (2018)
SYNPCC7002_A2771	SySSADH_2	Other SSADHs	*Synechococcus* sp. PCC 7002	WP_012308363	454	PDB: 4IT9	NADP^+^(H)	Dimer	Park and Rhee (2013) and Zhang and Liu (2015)
yneI	StSSADH	Other SSADHs	*Salmonella typhimurium*	WP_000178397	462	PDB: 3ETF	NAD^+^(H) more than NADP^+^ (H)	Dimer (Tetramer at high concentration)	Zhang and Liu (2015) and Zheng et al. (2013)
ALDH5F1	SSADH1	ALDH5 (Human‐like SSADHs)	*Arabidopsis thaliana*	AAF23590	528	AFDB: AF‐Q9SAK4‐F1	NAD^+^(H)	Tetramer	Busch and Fromm (1999) and Toyokura et al. (2011)
all3556	ApSSADH	Other SSADHs	*Anabaena* sp. PCC7120	BAB75255	455	AFDB: AF‐Q8YR92‐F1	NADP^+^(H)	Dimer	Wang et al. (2018)
gabD	CySSADH	Other SSADHs	*Cyanothece* sp. ATCC51142	ACB53576	455	AFDB: AF‐B1WSJ7‐F1	NADP^+^(H)	Dimer	Xie et al. (2020)
gabD	EcGabDSSADH	ALDH5 (Human‐like SSADHs)	*Escherichia coli*	AAC36831	482	PDB: 3JZ4	NADP^+^(H)	Tetramer	Ahn et al. (2010), Cozzani et al. (1980), and Donnelly and Cooper (1981)
sad yneI	EcYnelSSADH	Other SSADHs	*Escherichia coli*	AAC74598	462	AFDB: AF‐P76149‐F1	NAD^+^(H) more than NADP^+^ (H)	Dimer	Donnelly and Cooper (1981) and Fuhrer et al. (2007)
UGA2	ScSSADH	ALDH5 (Human‐like SSADHs)	Saccharomyces cerevisiae	CAA84943	497	AFDB: AF‐P38067‐F1	NAD^+^(H)	Tetramer	Cao et al. (2014) and Langendorf et al. (2010)
ssadh	LcSSADH	ALDH5 (Human‐like SSADHs)	*Lucilia cuprina*	CAP78905	507	AFDB: AF‐B0JFD4‐F1	NAD^+^(H)	NA	Rothacker et al. (2008)
ssadh	CfSSADH	ALDH5 (Human‐like SSADHs)	*Ctenocephalides felis*	CAP78907	509	AFDB: AF‐B0JFD6‐F1	NAD^+^(H)	NA	Rothacker et al. (2008)
ASPNIDRAFT_57046	AnSSADH	ALDH5 (Human‐like SSADHs)	*Aspergillus niger*	EHA20532	531	AFDB: AF‐G3Y8S4‐F1	NAD^+^(H)	NA	Kumar et al. (2015)
Aldh5a1	MmSSADH	ALDH5 (Human‐like SSADHs)	*Mus musculus*	BAC35105	523	AFDB: AF‐Q8BWF0‐F1	NAD^+^(H)	Tetramer	Rivett and Tipton (1981)
AGABI1DRAFT_113228	AgbSSADH	ALDH5 (Human‐like SSADHs)	*Agaricus bisporus Lge*	EKM79984	528	AFDB: AF‐K5VZN4‐F1	NAD^+^(H) more than NADP^+^ (H)	NA	Baldy (1977)
lmo0913	LmSSADH	ALDH5 (Human‐like SSADHs)	*Listeria monocytogenes*	CAC98991	488	AFDB: AF‐Q8Y8I9‐F1	NA	NA	Feehily et al. (2013)

Abbreviations: AF, predicted with AlphaFold2 in this work by using Colabfold scripts (https://github.com/sokrypton/ColabFold); AFDB, obtained from AlphaFold‐EBI protein structure database (https://alphafold.ebi.ac.uk/) with the ID reported; PDB, obtained from Protein Data Bank (https://www.rcsb.org/) with the ID reported; NA, not available.

^a^ The classification of ALDH families is based on Vasiliou and Nebert (2005), while the distinction between “Human‐like” or “Other” SSADHs is based on the evolutionary analyses presented in this study.

^b^ Source of protein structures used for building the alignment used for the phylogeny in Figure 1.

In the present work, we initially carried out an evolutionary investigation of the SSADH class of enzymes, determining the phylogenetic relationships of hSSADH and other SSADHs or human ALDHs and highlighting the phylogenetic traits conserved in human‐like SSADHs. Second, a deep characterization of the spectroscopic and kinetic properties of WT hSSADH was accomplished and the molecular basis for catalytic competence was proposed by investigating spectroscopic and kinetic features of variants of ionizable active site residues suggested to be highly conserved in human‐like SSADHs. Overall, we suggested that, at least for hSSADH, efficient catalysis is controlled by a network of active site residues in contact with the two catalytic ones (Cys340 and Glu306). This analysis could ultimately pave the way for the interpretation of the loss‐of‐function of SSADH pathogenic variants affecting not only the active site but also the intricate network of interactions shaping it.

## 2 RESULTS AND DISCUSSION

### 2.1 Known SSADHs belong to two deeply divergent evolutionary groups with differences in the oligomerization state and in the network of conserved active site residues

Given the large number of available SSADH and SSADH‐like sequences, an evolutionary‐bioinformatic analysis could provide relevant information regarding residues responsible for oligomeric assembly, active site organization, and cofactor preference of hSSADH. This information could also be valuable in identifying the molecular rationale of SSADH activity in pathogenic variants. Therefore, we studied the evolution of biochemical and structural properties of characterized SSADHs in the sequence space of the ALDH superfamily.

**FIGURE 1:**
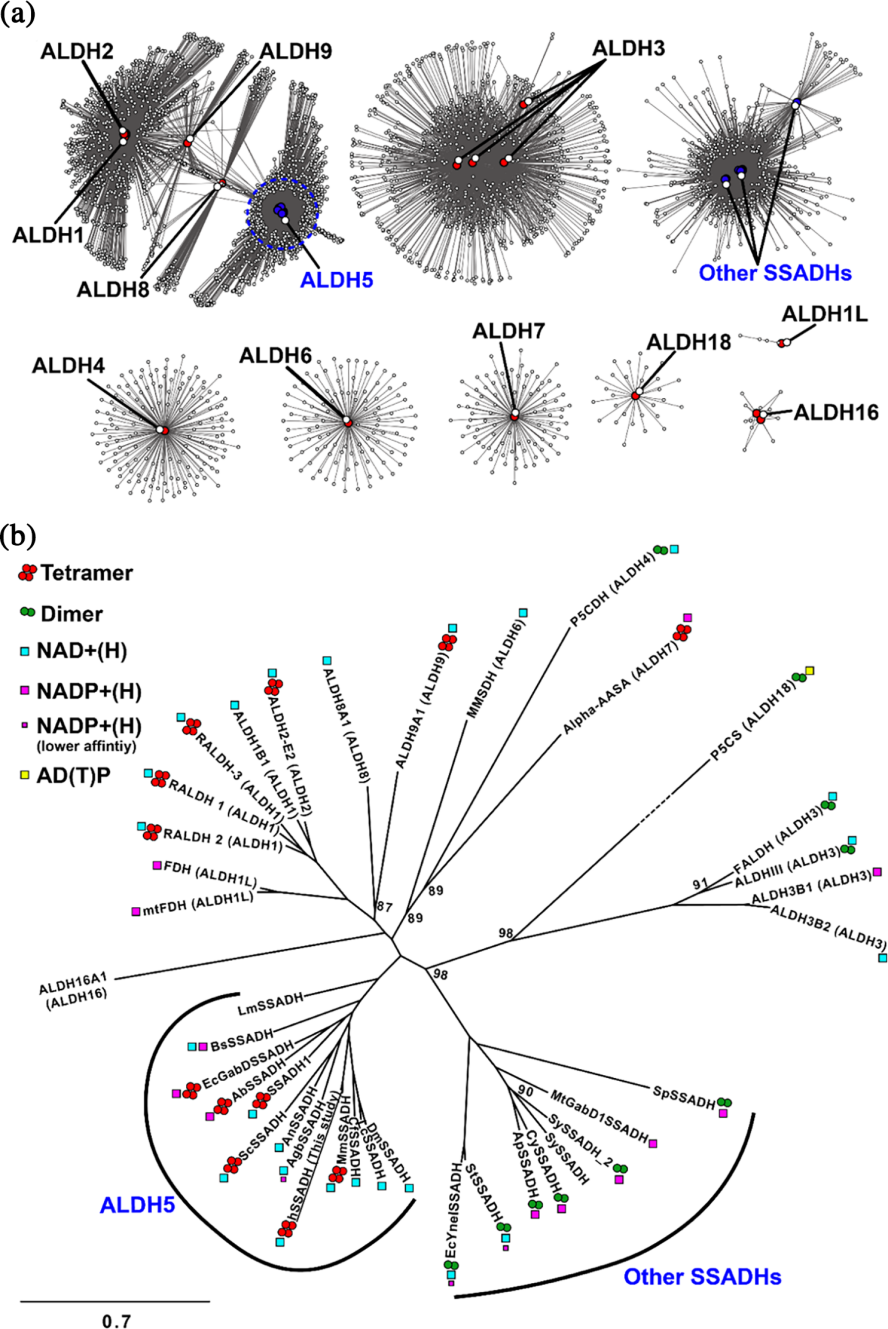
Diversity and evolution of SSADHs within ALDH superfamily and maximum likelihood unrooted phylogenetic tree of characterized SSADHs and ALDHs coded by human genes. (a) Sequence similarity networks (SSNs) of ALDH superfamily homologous to human ALDHs or characterized SSADHs from non‐human organisms. See the text for info on how the sequences were collected. The edge‐weighted spring embedded layout with respect to the edge weights was used for visualization. A dashed blue circle highlights the region of the sequence space encompassing nodes that have a minimum of 45% sequence identity to hSSADH. (b) See Table 1 for details on sequence identifiers. The structures used for aligning the sequences are from PDB, AlphaFold‐EBI protein structure database (https://alphafold.ebi.ac.uk/) or predicted with AF2; info reported in Table 1. The branch length is proportional to the expected substitution rate, according to the reference bar. Only nodes with bootstrap support <97 are labeled.

Sequence similarity networks (Figure 1a) built at a minimum 40% global pairwise sequence identity (a threshold commonly used for discriminating between ALDH families; Vasiliou and Nebert 2005) suggest that SSADHs belong to two different paralogue families, referred in this study as the *ALDH5*, which includes hSSADH, and *Other SSADHs*. The first one includes SSADHs from Eukaryotes (including hSSADH) and from some bacterial species (*Escherichia coli*, *Acinetobacter baumanii*, *Listeria monocytogenes*, *Bacillus subtilis*; Table 1), usually annotated as the “gabD” paralogue gene of *E. coli* (Fuhrer et al. 2007), while the second includes only bacterial SSADHs usually annotated as the “yneI” paralogue gene of *E. coli*.

**FIGURE 2:**
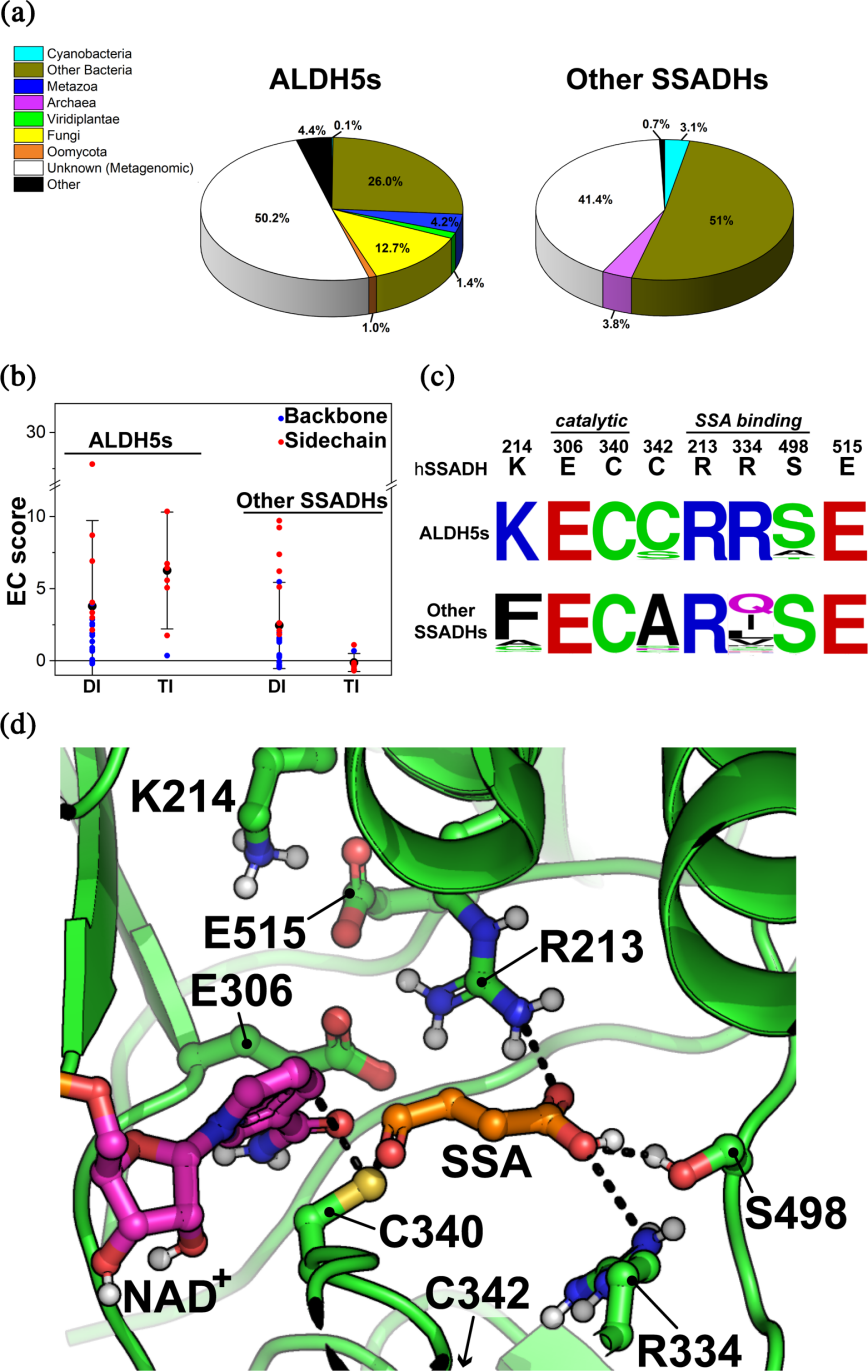
Evolutionary comparison of representative sequences from *ALDH5* and *Other SSADH* families. (a) Taxonomy distribution of the source organisms for each SSADH family. (b) Box plot of evolutionary coupling (EC) scores (see section 4) for each residue pair predicted to interact at oligomeric interfaces in hSSADH simulations (Table S1). DI, residue pairs at dimeric interface; TI, residue pairs at tetrameric interface; Backbone, EC scores of residue pairs interacting by backbone atoms; Sidechain, EC scores of residue pairs interacting by sidechain atoms. (c) Frequency logo of catalytic and titratable residues of hSSADH within representative sequences of the two SSADHs families. (d) hSSADH active site with the catalytic Cys340 manually deprotonated (PDB ID: 2W8O). NAD^+^ and SSA were fully flexible docked with DynamicBind v.1.0 (plDDT_SSA_ ≈ 0.75, plDDT_NAD_^+^ ≈ 0.69; see section 4) and visualized in ball‐and‐sticks together with catalytic and titratable active site residues. Black dashed lines connect the sulfur atom of the catalytic Cys340 to the reactive carbon centers of NAD^+^ and SSA.

The phylogenetic analysis confirmed that both SSADH families are monophyletic, with *ALDH5s* closely related to other tetrameric ALDHs, while *Other SSADHs* members are related to dimeric ALDHs (Figure 1b). We retrieved representative sequences of each SSADH family (2191 for the *ALDH5* and 3011 for the *Other SSADH*) to gain insight on this structural difference and additional features, through the analysis of taxonomy, coevolution, and the conservation of critical active site residues. A clear difference in the taxonomic distribution is evident: only the *ALDH5* family contains sequences from Eukaryota (i.e., Metazoa, Fungi, Viridiplantae), while the *Other SSADH* family essentially contains members from Eubacteria and Archea (Figure 2a). The large fraction of taxonomically unassigned metagenomic samples in both families was not considered in this analysis.

We used direct coupling analysis on alignments of representative sequences of both families to analyze the evolutionary coupling scores (EC scores, corresponding to co‐evolutionary signals). Residues that formed stable non‐covalent interchain contacts over molecular dynamics (MD) simulations replicates of the hSSADH tetramer were considered (Table S1 and Figure S1). While there is an equal number of high EC scores between residues corresponding to dimeric contacts in both *SSADH* and *ALDH5* families, high EC scores corresponding to residues at the tetrameric interface were inferred only for the *ALDH5* family (Figure 2b). As expected, interfacial residues showed a high positive EC score usually only when interacting by side chain atoms, while residue pairs interacting through backbone atoms showed very low EC scores, both at the dimeric and tetrameric interface. We further tested if the presence of differently distributed co‐evolutionary information in the alignments could also impact the Alpha‐Fold (AF) template‐free modeling capacity of a tetrameric assembly of hSSADH, using either the *ALDH5* or the *Other SSADH* alignments as inputs. Predicted interfacial structural quality scores (iPTM score) > 0.75 usually result in correctly modeled interfaces. We obtained an iPTM > 0.9 at dimeric interfaces for most of the models produced using both alignments. On the contrary, the iPTM scores at tetrameric interfaces were >0.9 only for models that used the alignment of sequences from *ALDH5* family, while it was significantly lower (<0.437) for most of the models produced from sequences from the *Other SSADH* family. Overall, the performed analyses suggested that (i) during the evolution of interfacial residues, a strong co‐evolutionary constraint is present when interfacial interactions need to preserve amino acid type (reflected by its side chain), while residues interacting through backbone are apparently less constrained, as also observed in Hockenberry and Wilke (2019); (ii) the co‐evolutionary signals at the tetrameric interface suggest that most, if not all, *ALDH5s* are tetrameric, while *Other SSADHs* are mostly if not all, dimeric. Importantly, several kinetic and modeling studies on the SSADH reaction mechanism were performed on bacterial SSADHs belonging to the dimeric family (SySSADH and StSSADH; Table 1). This highlights the need to perform a detailed functional investigation on the human enzyme, for which significant differences are expected.

Interestingly, residues Arg173, Gly176, and Gly533 at the dimeric and/or tetrameric interface (Table S1) are substituted in SSADH deficiency (due to missense mutations in *ALDH5A1*) and their pathogenicity was associated with a folding/oligomerization failure (Tokatly Latzer et al. 2023c).

Previous studies identified catalytically important residues of hSSADH (Kim et al. 2009): the catalytic pair Cys340 and Glu306, and the residues Arg213, Arg334, and Ser498, essential for SSA binding. By inspecting the active site of the crystal structure of the oxidized form of hSSADH (PDB ID: 2W8O; Figure 2d) two additional titratable residues, Lys214 and Glu515, interacting with Glu306, were identified. These residues could participate in modulating the protonation state of the latter residue. Among the conserved residues, it is interesting to note that only Cys340, Glu306, Glu515, Arg213, and Ser498 are conserved among all SSADHs, while Cys342, Lys214, and Arg334 are conserved only in the *ALDH5* family (Figure 2c). This indicates that the SSADHs which possess a single cysteine at the active site and the ones that have two active site cysteine residues (Kim et al. 2009) belong to two distinct families and, besides a similar general mechanism and the possibility to oxidize the same substrate, they may display profound differences in their kinetic properties and a diverse substrate preference consistent with the usually broad substrate specificity observed in *Other SSADHs* (Table 1). Despite such distinct patterns of conservation of the active site, the cofactor preference is not phylogenetically clustered (Figure 1b). All eukaryotic *ALDH5s* have high affinity only for NAD^+^, while prokaryotic *ALDH5* and most of *Other SSADHs* prefer NADP^+^ or have promiscuous affinity for both cofactors. Since a switch between NAD^+^ and NADP^+^ preference requires only a few amino acid substitutions (Jang et al. 2014; Yuan et al. 2013), we suggest that this feature is not phylogenetically grounded, but it has been tweaked with respect to the type of cofactor used in redox reactions in different taxa and subcellular compartments (i.e., in eukaryotic mitochondria NAD^+^ is used and NADP^+^ is absent; Stein and Imai 2012).

Based on the in silico information about differences between SSADHs belonging to different phylogenetic groups, we designed a detailed investigation of the main structural and functional properties of hSSADH.

### 2.2 Catalysis of purified recombinant hSSADH proceeds through an ordered bi–bi mechanism and is regulated by SSA concentration

The stable and active tetrameric hSSADH has been expressed and purified as described (Didiasova et al. 2024) and its spectroscopic properties have been determined (for details see Results section in Data S1, Table S2, and Figures S2a–d, S3, and S4). Since the kinetic features of hSSADH have not been previously investigated in detail, we carried out a functional characterization.

The determination of the kinetic parameters of the reaction catalyzed by hSSADH was a complex task due to the partial non‐competitive inhibition exerted by the substrate SSA (Figure S5). Overall, data indicate a behavior compatible with an ordered bi–bi mechanism, with NAD^+^ binding first to the enzyme (Results section in Data S1, Figures S6 and S7, and Tables S3 and 2), a kinetic mechanism shared with several aldehyde dehydrogenases (Munoz‐Clares and Casanova‐Figueroa 2019). We determined the most reliable estimate for *k*_cat_ (about 160–170 s^−1^) at 25°C, while *K*_*m*_ values for both cosubstrates and *K*_*i*_ for SSA were determined at a lower temperature (14°C) to slow down the reaction rate (Results section in Data S1 and Table 2). These analyses resulted in the determination of *K*_*m*SSA_ = 1.2 ± 0.2 μM and *K*_*m*NAD+_ = 31 ± 5 μM, *K*_*i*SSA_ = 13 ± 3 μM. Table 2 summarizes the values of kinetic parameters determined under different experimental conditions to reach the best estimates.

**TABLE 2:** Determination of the kinetic parameters of the reaction of hSSADH by fitting data to different models.

		From Dixon plots (25°C)	From Equation (3) (25°C)	From Equation (4) (25°C)	From Equation (5) (25°C)	From Equation (5) (14°C)
Subinhibitory (SSA)	*k*_cat_	80 ± 10 s^−1^	87 ± 4 s^−1^			
	*K*_*m*SSA_	0.6 ± 0.2 μM	1.5 ± 0.2 μM			
	*K*_*m*NAD+_	14 ± 8 μM	36 ± 5 μM			
Inhibitory (SSA)	*k*_cat_			165 ± 23 s^−1^		
	*K*_*m*SSA_			–		
	*K*_*m*NAD+_			32 ± 12 μM		
	*K*_*is*_			5 ± 3 μM		
	*K*_*ix*_			84 ± 12 μM		
	*K*_*ii*_			7 ± 2 μM		
Full range (SSA)	*k*_cat_				166 ± 33 s^−1^	26 ± 2 s^−1^
	*K*_*m*SSA_				4 ± 1 μM	1.2 ± 0.2 μM
	*K*_*m*NAD+_				84 ± 23 μM	31 ± 5 μM
	*K*_*i*SSA_				9 ± 3 μM	13 ± 3 μM
	*b*				0.030 ± 0.008	0.06 ± 0.01

**SCHEME 3:**
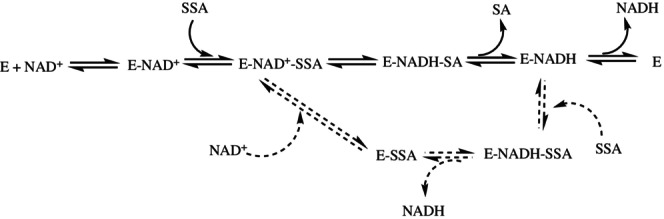
Proposed reaction mechanism for hSSADH. NAD^+^ binds first to the enzyme followed by SSA with the formation of the ternary complex. This then releases the SA and NADH products (straight lines). At high SSA concentrations, a partial non‐competitive inhibition exerted by SSA occurs, with the formation of a partial inhibitory ternary complex E‐SSA‐NADH. The reduced cofactor is then replaced by NAD^+^ to regenerate the catalytically competent ternary complex E‐SSA‐NAD^+^ (dashed lines). This outlined mechanism is based on extensive investigations previously reported for other aldehyde dehydrogenases that show a substrate (aldehyde) partial inhibition behavior similar to that of hSSADH (Munoz‐Clares and Casanova‐Figueroa 2019).

Given the competitive inhibition of NADH with respect to NAD^+^ (see Results section in Data S1), the partial non‐competitive component of the SSA inhibition could be due to its combination in a dead‐end fashion with the complex E‐NADH (Scheme 3), in analogy to what already observed for other aldehyde dehydrogenases with partial substrate inhibition (Munoz‐Clares and Casanova‐Figueroa 2019).

It should be noted that values of Michaelis–Menten and inhibition constants for SSA are in a low micromolar range. Thus, the effects exerted by the aldehyde could be relevant even at physiological concentrations. In addition, hSSADH exhibits a turnover rate (see above) far exceeding the values previously reported (Chambliss et al. 1995; Chambliss and Gibson 1992; Kang et al. 2005). As an example, rat SSADH exhibits a lower *k*_cat_ (Murphy et al. 2003), as well as SSADHs present in various organisms (Cao et al. 2014; Jang et al. 2014; Park et al. 2014; Park and Rhee 2013; Zheng et al. 2013), with the exception of the NADP^+^ dependent *Acinobacter baumanii* SSADH (Phonbuppha et al. 2018). This discrepancy is in line with the fact that previous kinetic analyses in those papers were carried out without considering the whole range of cosubstrate concentrations, as instead suggested for aldehyde dehydrogenases by Munoz‐Clares and Casanova‐Figueroa (2019).

**FIGURE 3:**
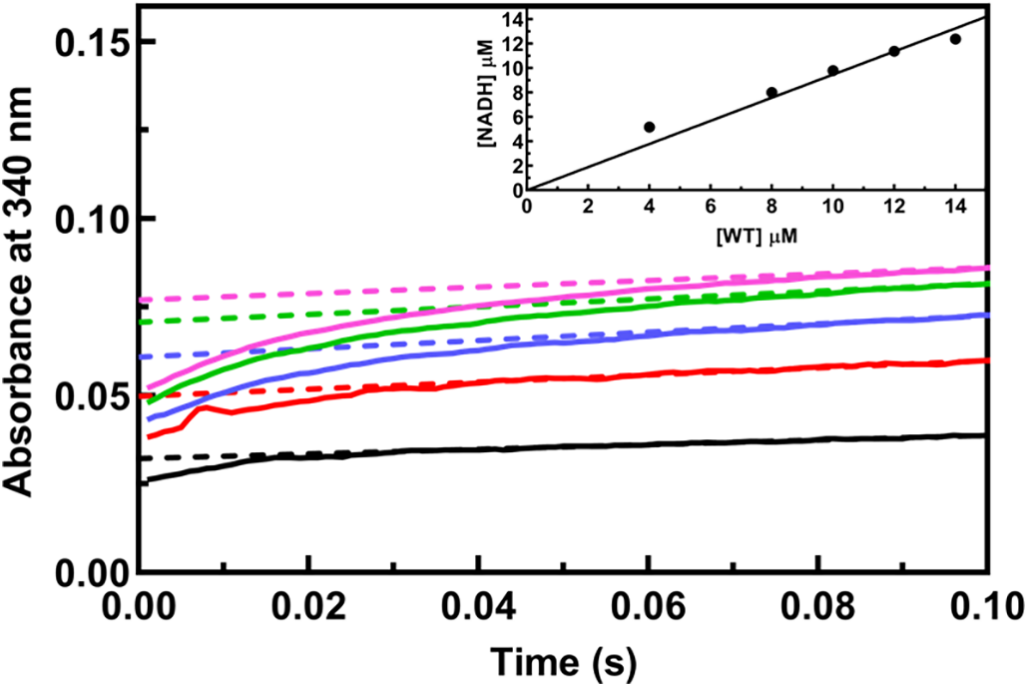
Burst kinetics and active site titration of hSSADH. Reactions were carried out at 20 μM SSA and 500 μM NAD^+^ by adding the following hSSADH concentrations: 4 μM (black), 8 μM (red), 10 μM (blue), 12 μM (green), and 14 μM (pink). The absorbance change at 340 nm was monitored using a stopped‐flow spectrophotometer at 4°C in 100 mM potassium phosphate buffer, 10 mM BME at pH 8. The inset shows the correlation of the concentration of NADH formed during the burst phase with hSSADH concentration.

Stopped‐flow pre‐steady state kinetics shows a burst of NADH formation in 15–20 ms before reaching the turnover steady‐state (Figure 3). This implies that hydride transfer from the hemiacetal to the cofactor is faster than the following steps of deacylation and product release. The amount of NADH produced in the burst phase linearly correlates with the enzyme concentration (Figure 3, inset), suggesting that all enzyme active sites fully accomplish the reaction. This differs from what reported in *Acinobacter baumanii* SSADH (Phonbuppha et al. 2018) where only a small percentage of active sites are active.

### 2.3 The SSADH‐NAD ^+^ complex stabilizes hSSADH and protects the catalytic Cys340

We carried out the spectroscopic characterization (by circular dichroism, absorbance and fluorescence emission) of WT hSSADH in the absence and in the presence of NAD^+^ to reveal spectral perturbation following the binding of the cofactor. This information is critical when dealing with variants (including the pathogenic ones) that could result in the alteration of cofactor binding both in terms of affinity and/or of microenvironment modifications.

**FIGURE 4:**
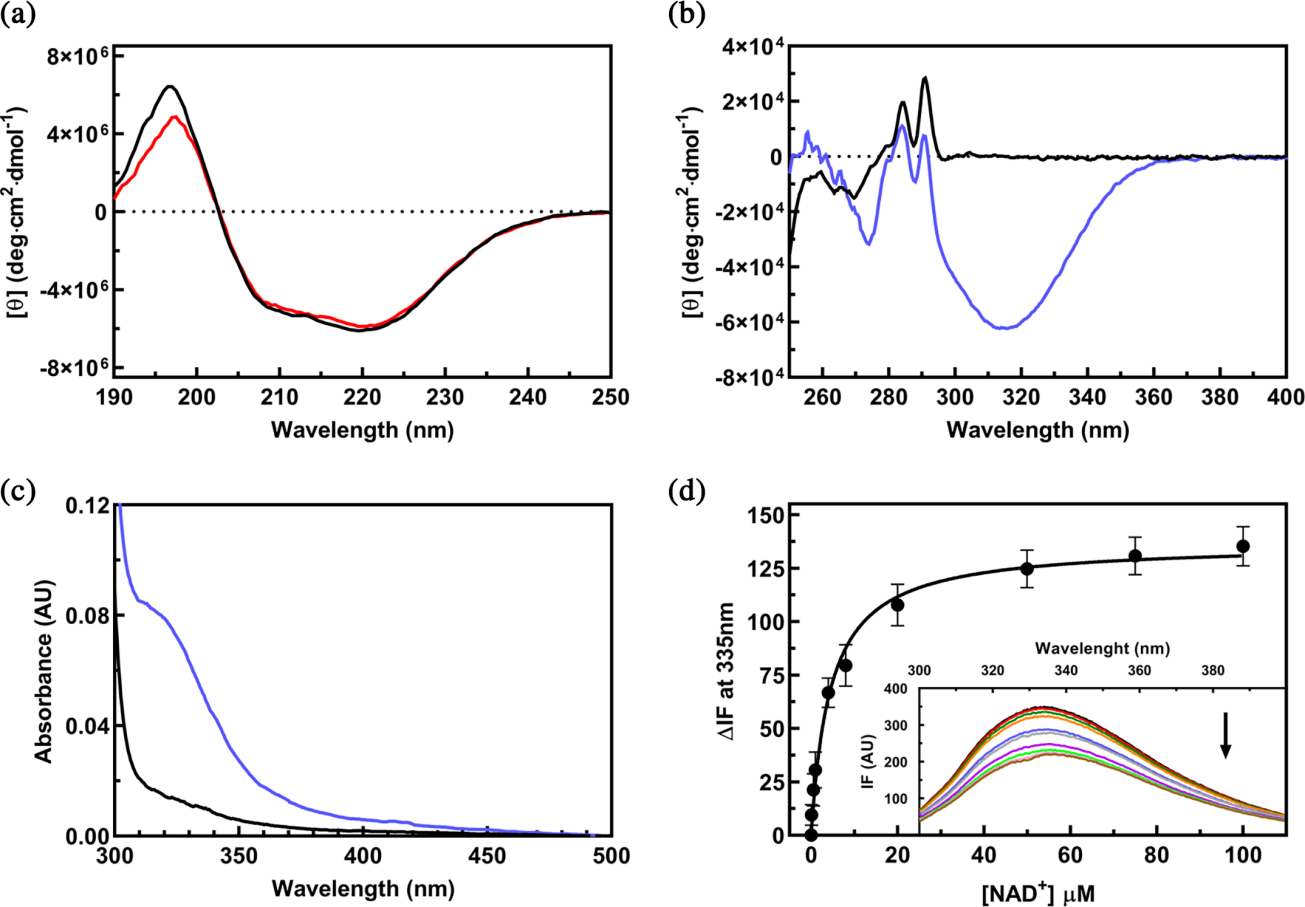
Spectral characterization of WT hSSADH. All spectra were collected in 100 mM potassium phosphate buffer, 10 mM BME at pH 8 at 25°C. (a) Far UV CD spectra of 2 μM (monomer concentration) WT hSSADH in the absence (black line) or presence of 100 μM NAD^+^ (red line). (b) Near UV–visible spectra of 18 μM WT hSSADH in the absence (black line) or presence (blue line) of 200 μM NAD^+^. (c) Absorbance spectra of 18 μM WT hSSADH in the absence (black line) or presence (blue line) of 200 μM NAD^+^. (d) Plot of the quenching in intrinsic fluorescence spectra of 0.1 μM WT hSSADH in the presence of different concentrations of NAD^+^. Data were fitted to a hyperbola equation to obtain the *K*_*D*_ value. Inset: Intrinsic fluorescence spectra of 0.1 μM WT hSSADH (black line) in the presence of different concentrations of NAD^+^ (0.1 μM, red; 0.5 μM, dark green; 1 μM, orange; 4 μM, blue; 8 μM, gray; 20 μM, purple; 50 μM, light green; 75 μM, pink; and 100 μM, brown). The arrow indicates the change at increasing cofactor concentrations.

The far UV circular dichroism (CD) spectrum of 2 μM hSSADH (the concentration is always reported as the monomer concentration, unless for kinetic assays since the active functional enzyme species is the tetramer) presents two negative (208 and 222 nm) and one positive (195 nm) bands typical of a high α‐helical content protein (Figure 4a). Deconvolution of the spectrum reveals a 29.7% of α‐helix and 29.9% of β‐strand structures content. Overall, these values are in agreement with the ones determined from the analysis of the crystal structure: 37% of α‐helix and 27.6% of β‐strand structures (Kim et al. 2009). The presence of 100 μM NAD^+^ produces minor modifications in the far UV signals with a modest decrease in α‐helix content (24.9%) and an almost identical β‐structure percentage (29.6%). This is reminiscent of the effects observed upon the binding of NAD^+^ to other dehydrogenases (Murtas et al. 2021).

Binding of NAD^+^ results in a ~4°C increase in *T*_*m*_, as determined by thermal denaturation at 222 nm by CD spectroscopy and by differential scanning calorimetry (DSC) (Table S4), suggesting that NAD^+^ binding confers higher thermal stability. On the other hand, the presence of a reducing agent does not seem to significantly influence thermal stability, suggesting that disulfide bonds do not play a major role in protein stabilization (see Results section in Data S1).

The near‐UV visible CD spectrum of 18 μM hSSADH shows two positive peaks at 284 and 291 nm, in addition to small negative signals in the region around 265 nm, attributable to the aromatic residues of the enzyme (Figure 4b). NAD^+^ binding (200 μM) leads to the appearing of a strong negative signal centered at 315 nm (not due to free NAD^+^ in solution) and to a modification in the region of the aromatic amino acids of SSADH (Figure 4b). These spectral changes are suggestive of a binding, resulting in the positioning of the cofactor in an asymmetric microenvironment. The formation of hSSADH‐NAD^+^ complex is also observed by absorbance spectral analysis where binding of NAD^+^ results in the formation of a broad shoulder at 320 nm (Figure 4c). This absorbance behavior, reported for some SSADH enzymes in the presence of their cofactor, was interpreted as the formation of a covalent complex (Paladkong et al. 2022; Park and Rhee 2013). Finally, the intensity of intrinsic protein fluorescence (excitation at 295 nm, emission at 335 nm) is quenched in the presence of NAD^+^ due to internal energy transfer, thus further supporting a tight binding of the cofactor to the enzyme (Figure 4d, inset). Altogether, NAD^+^ binding can be clearly followed by CD, absorbance or fluorescence spectroscopy, and results in a thermal stabilization of hSSADH.

The equilibrium dissociation constant of NAD^+^ has been measured by evaluating the decrease of the emission band at 335 nm (*λ*_exc_ 295 nm) following the addition of increasing concentrations of NAD^+^ to 0.1 μM hSSADH (for details see Materials and methods section in Data S1). The plot of the quenching variations as a function of NAD^+^ concentration was fitted to a hyperbola equation resulting in a *K*_*D*_ = 4.5 ± 0.8 μM (Figure 4d). The fact that SSADH could covalently and reversibly bind NAD^+^ (independently of SSA) has been proposed to be a protective mechanism to avoid hyperoxidation of the catalytic active site Cys340, as suggested not only for a bacterial *Acinobacter baumanii* SSADH, but also for other aldehyde dehydrogenases (Munoz‐Clares et al. 2017; Phonbuppha et al. 2018). The cofactor binding should proceed through a nucleophilic attack of the catalytic Cys thiolate to the C4 atom of the NAD^+^ nicotinamide ring. This covalent bond reversibly dissociates in the presence of SSA (Phonbuppha et al. 2018). This can be the case also for hSSADH and is corroborated by the higher affinity of SSA with respect to NAD^+^. Thus, the formation of the binary complex between the enzyme and the NAD^+^ cofactor could be regarded as a shielding strategy to limit the accessibility to the catalytic Cys340 by oxidizing agents.

### 2.4 Acid–base groups govern catalysis and NAD ^+^ equilibrium binding

**FIGURE 5:**
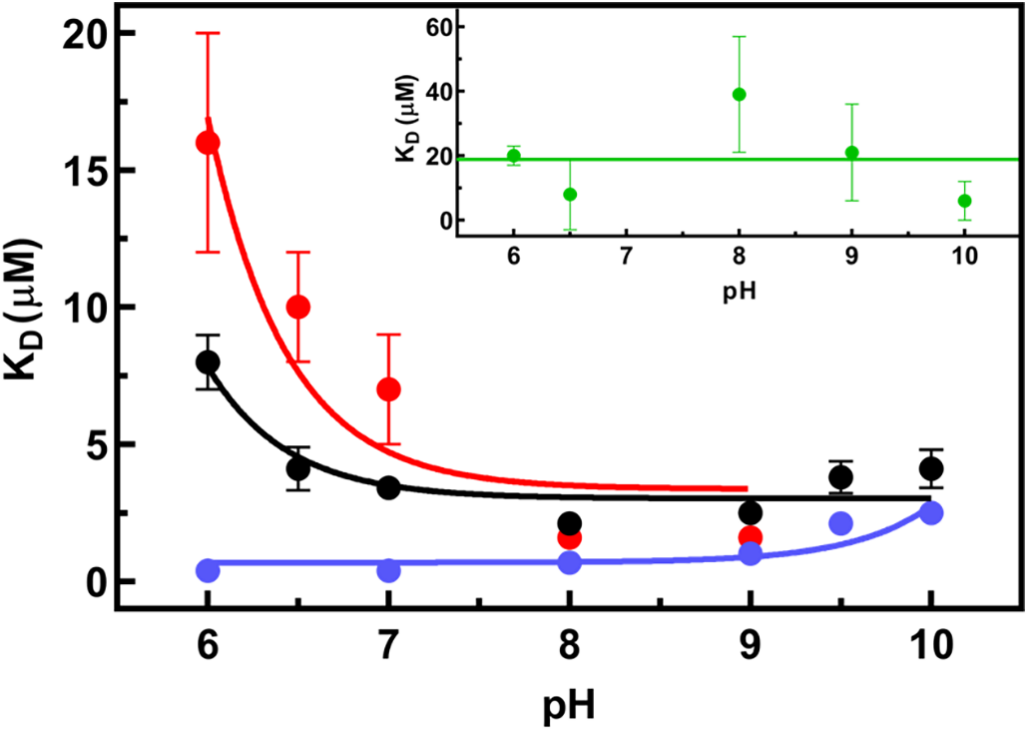
Dependence of *K*_*D*NAD+_ on pH. All measurements were performed in 50 mM BTP and 10 mM BME at the indicated pH values. At each pH value, *K*_*D*NAD+_ was obtained by measuring the quenching of intrinsic fluorescence determined by exciting 0.1 μM WT hSSADH (black), 0.1 μM Lys214Ala (blue), 0.1 μM Glu515Ala (green), and 0.4 μM Glu306Ala (red) at different NAD^+^ concentrations (from 0.1 to 100 μM) and fitting the data to a hyperbolic equation. The pK_a_ determination was obtained by fitting the resulting *K*_*D*_ values to Equation (6).

We determined the value of *K*_*D*_ for NAD^+^ as a function of pH in 50 mM Bis‐Tris‐Propane (BTP) given its wide buffering range. At pH 8 the measured *K*_*D*_ value is nearly similar (2.1 ± 0.4 μM) to the one evaluated in potassium phosphate buffer. The dissociation constant for NAD^+^ decreases at increasing pH values, indicating an affinity increase, possibly due to the deprotonation of a residue with a pK_a_ of 6.36 ± 0.06 (Figure 5).

**FIGURE 6:**
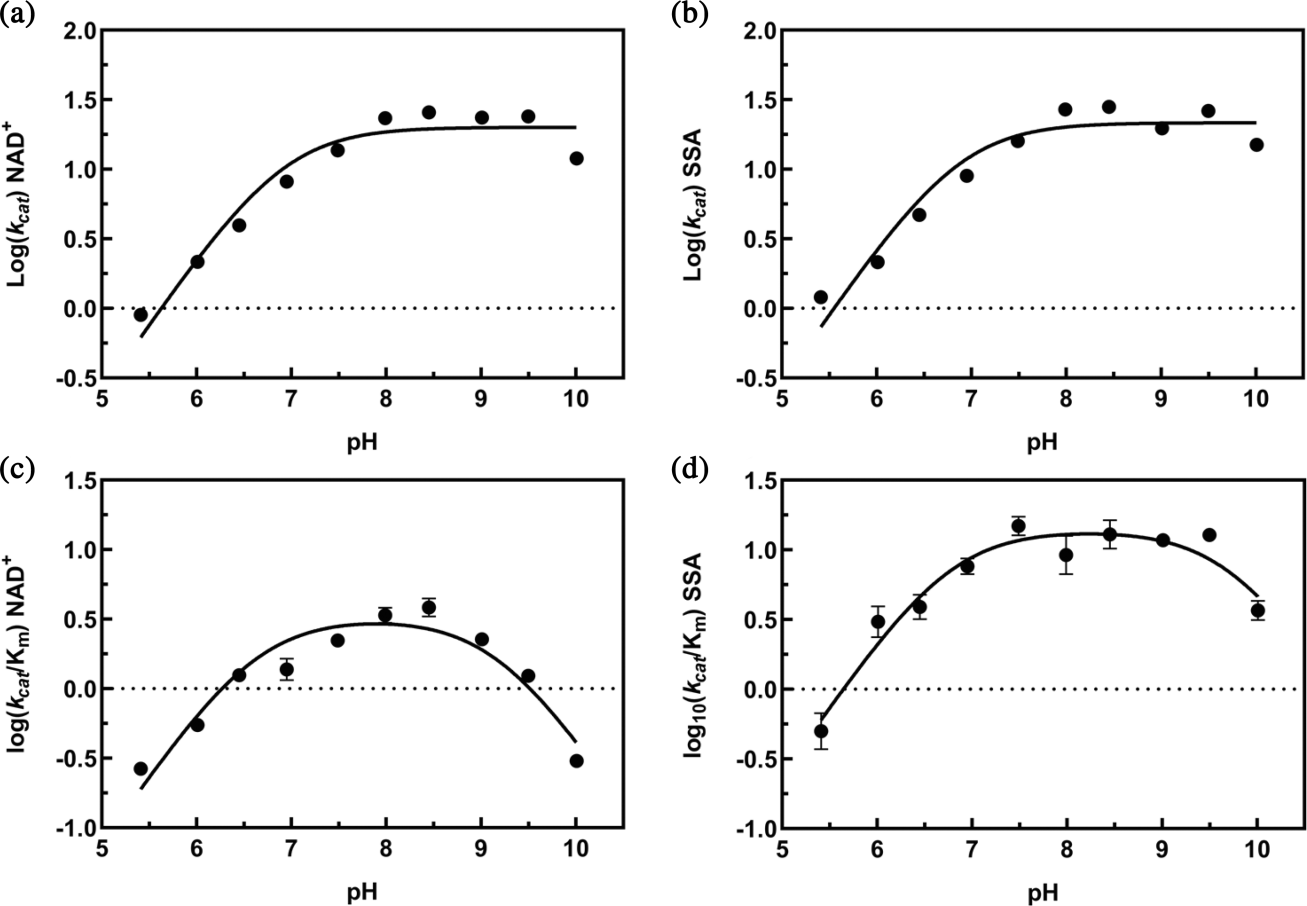
Dependence of kinetic parameters on pH. Kinetic parameters were determined at the following pH values: 5.4, 6.0, 6.5, 7.0, 7.5, 8.0, 8.5, 9.0, 9.5, and 10.0. All measurements were performed in 50 mM BTP and 10 mM BME at the indicated pH values. Log *k*_cat_ for NAD^+^ (a) and for SSA (b) versus pH, as well as Log *k*_cat_/*K*_*m*_ for NAD^+^ (c) and for SSA (d) versus pH were determined and fitted to Equations (7) and (8), respectively.

Furthermore, we determined the kinetic parameters of the reaction as a function of pH for both cosubstrates (see section 4). The resulting kinetic parameters were plotted as log *k*_cat_ or log *k*_cat_/*K*_*m*_ for both cosubstrates versus pH. The plots of log *k*_cat_ for both SSA and NAD^+^ exhibit an increase in the acidic range which reaches a plateau above pH ~8 (Figure 6a,b). Data were fitted to Equation (7) yielding a pK_a_ value of ~6.5–7 that could be attributed to the protonation state of the enzyme–cosubstrates catalytic complex. Since the same pK_a_ is also found in the log *k*_cat_/*K*_*m*_ plot (fitted to Equation (8) and Figure 6c,d), it can be reasonably attributed to an enzyme residue involved in catalysis. The second pK_a_ (~9.5) obtained in the bell‐shaped dependence of log *k*_cat_/*K*_*m*_ plot could be attributed to either an enzyme or a substrate group involved in catalysis or binding. All pK_a_ values are reported in Table 3. Interestingly, the pK_a_ affecting NAD^+^ affinity is in the same range as the acidic catalytic pK_a_.

**TABLE 3:** pKa values for the kinetic parameters of the reaction.

	pKa1	pKa2
log *k*_cat_ SSA	6.9 ± 0.1	–
log *k*_cat_/*K*_SSA_	6.8 ± 0.1	9.7 ± 0.2
log *k*_cat_ NAD^+^	6.9 ± 0.1	–
log *k*_cat_/*K*_NAD+_	6.6 ± 0.2	9.2 ± 0.2

*Note*: Experiments were carried out in 50 mM BTP at pH values from 5.5 to 10 at 25°C.

Attributing the obtained pK_a_ values to specific enzyme groups is rather challenging. The pK_a_ in the basic region, visible only in the *k*_cat_/*K*_*m*_ plots, most likely reflects a basic group of the enzyme that needs to be protonated to efficient catalysis and/or binding. Two basic residues (Arg213 and Arg334) are present at the active site and are involved, together with Ser498, in an electrostatic/H‐bonding network with the carboxy moiety of SSA (Kim et al. 2009). Arg213 is at 4.1 Å from the carboxy group of the catalytic Glu306 in the reduced SSADH form, and at 4.2 Å from the same group in the structure solved in the presence of SSA (Figure 2d). These distances are compatible with a strong electrostatic interaction. Thus, we suggest that the basic pK_a_ could be attributed to one or both of these Arg residues, engaged in an extensive network at the active site, playing a crucial role in binding the SSA substrate.

The attribution of the acidic pK_a_ of ~6.5–7, present in the plots of the dependence of the kinetic parameters and the NAD^+^ *K*_*D*_, needed more consideration.

First, based on literature, bioinformatic predictions (Table S5) and knowledge regarding this class of enzymes, we proposed the catalytic Glu306 as the most probable candidate. Its carboxylate is positioned at 3.8 Å from the catalytic Cys340 to accomplish its acid–base role in catalysis (deprotonation and reprotonation steps; Scheme 2) and forms the thiolate group able to perform a nucleophilic attack on a suitable electrophile (either NAD^+^ or the carbonyl of SSA). Moreover, the bacterial *Acinobacter baumanii* SSADH presents a kinetic and titration pK_a_ of ~7.5, suggested to be responsible for the formation of the thiolate state of the catalytic cysteine (Phonbuppha et al. 2018). However, in that paper, the observed pK_a_ was attributed to either the catalytic glutamate or to the catalytic cysteine itself (Phonbuppha et al. 2018) without discriminating among them.

Considering this conflicting attribution, reinforced by the fact that for most of the aldehyde dehydrogenases the catalytic cysteine residue is reluctant to deprotonation (Munoz‐Clares et al. 2017), we deepened our investigation. The prediction of pK_a_ values of cysteine residues is subject to potential overestimation with a variance of >2 pK_a_ units (Awoonor‐Williams et al. 2023). This depends on the fact that the actual pK_a_ of catalytic cysteine residues could be decreased by the network of H‐bonds established by the sulfur atom and/or by amide nitrogen of the nearby peptide bonds (Marchal and Branlant 1999). A mutational study on the bacterial *A. baumanii* SSADH suggested that the second Cys (of the CXC motif; Munoz‐Clares et al. 2017) could be responsible for lowering the pK_a_ of the catalytic cysteine (Paladkong et al. 2022). However, hSSADH Cys342Ala variant was shown to catalyze the dehydrogenase reaction efficiently and even more rapidly at the same pH value as the WT (Kim et al. 2009), ruling out that, at least in these experimental conditions, Cys342 may play a major role. Notably, the microenvironment of Cys340 shows that a salt bridge between Lys214 and Glu515 causes the carboxylate of the latter residue to be in proximity to the carboxylate of Glu306 (Figure 2d). Considering that Lys214 is highly conserved in ALDH5 family, it could be essential in maintaining the correct orientation of the Glu515 side‐chain to tackle Glu306, and, in turn, Cys340.

### 2.5 The active site hSSADH variants reveal the structural determinants for the interconnected bond network ensuring efficient catalysis

A mutagenic study was performed on the catalytic residues and the above listed second‐shell titratable active site residues of hSSADH, to define which ones could be directly or indirectly associated with the acidic pK_a_ value(s) observed in both hSSADH kinetics and in the *K*_*D*NAD+_ pH profiles.

Cys340Ala, Cys340Ser, Glu306Ala, Glu306Gln, Glu515Ala, Glu515Gln, and Lys214Ala variants have been expressed, purified, and characterized, showing no relevant structural changes or microenvironment alterations with respect to the WT (Results section in Data S1, Tables S6 and S7, and Figure S8a–c).

Substitutions of first‐shell catalytic residues Glu306 and Cys340 result in almost inactive variants (≤0.1% residual activity in comparison to the WT). In addition, Cys340 variants were unable to bind NAD^+^, while Glu306 variants displayed a different behavior: Glu306Ala presents a strongly reduced affinity for the cofactor (>10‐fold increased *K*_*D*NAD+_), while the conservative Glu306Gln has a cofactor affinity similar to that of the WT (4.3 ± 0.4 μM vs. 4.5 ± 0.8 μM, respectively) (Table S6). The fact that NAD^+^ still binds to Glu306 variants could reflect the presence of a small proportion of Cys340 thiolate that is however unable to undergo the acid/base catalysis.

Substitutions of second‐shell residues Lys214 and Glu515 resulted in variants retaining only 1.2% and 1.3% residual activity for Lys214Ala and Glu515Ala, respectively. The conservative substitution Glu515Gln had only a moderate effect on activity (residual activity ≈66%). Both residues Lys214 and Glu515 (Figure 2d) participate in the stabilization of the network of weak interactions with Cys340 and NAD^+^, thus playing an essential role in NAD^+^ binding and in the chemical step of catalysis.

**FIGURE 7:**
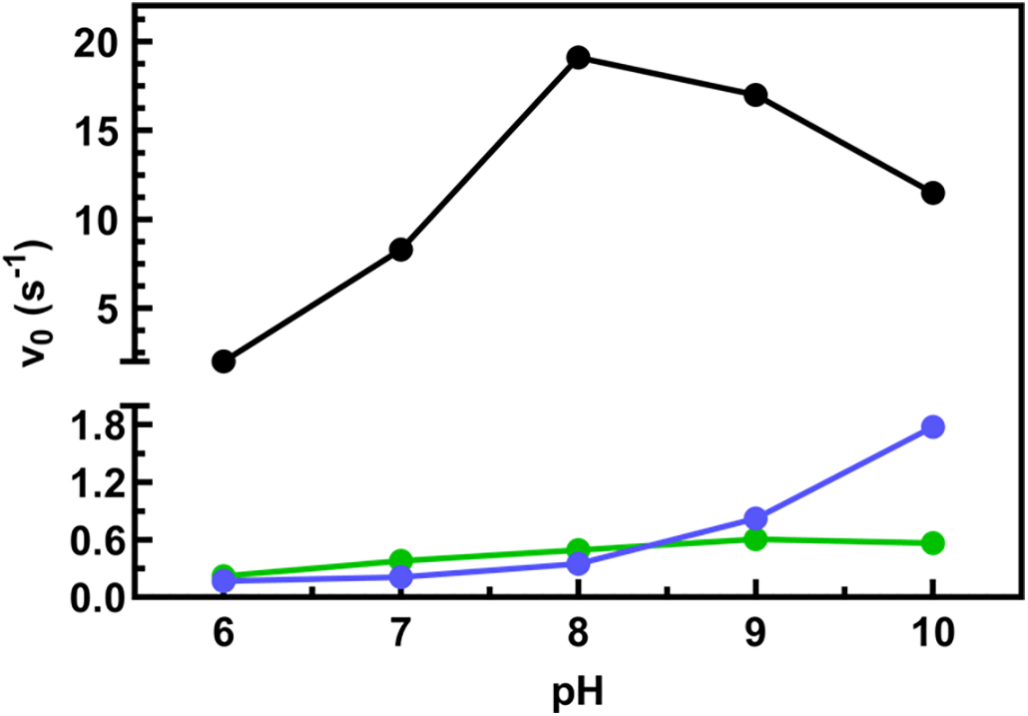
Dependence of activity on pH for active SSADH variants. Activity was determined by measuring the formation of NADH (see section 4 for details) obtained by incubating 8 nM WT (black), or 0.1 μM Glu515Ala (green), or 0.35 μM Lys214Ala (blue) with 10 μM SSA and 500 μM NAD^+^. The amount of NADH produced was expressed as micromol/sec/micromol of tetrameric enzyme (v_0_).

Ala‐substitutions result in the loss of the salt bridge between Lys214 and Glu515 and/or loss of H‐bonds essential to establish a crucial interaction with Glu306. It is conceivable that this perturbation could alter the pK_a_ of Glu306 side chain (Scheme 2). If the proton flow is impaired, catalysis proceeds slowly. Actually, for both variants, the initial reaction rates are insensitive to pH under the same assay conditions (10 μM SSA, 500 μM NAD^+^) (Figure 7) (with a slight increase for Lys214Ala at basic pH values), thus corroborating an effect on Glu306.

As for NAD^+^ binding, Lys214Ala showed an affinity higher than WT, possibly due to the increase in hydrophobicity of the active site pocket (the *K*_*D*NAD+_ for this variant was 2.5 ± 0.2 μM at pH 8). On the other hand, both variants at position Glu515 showed a large decrease (~4‐ to 9‐fold) in the affinity for cofactor (Table S6) under the same experimental conditions. This could indicate an alteration of the relative orientation of Cys340/nicotinamide ring, as suggested by spectroscopic signals.

The pH dependence of the affinity constant for the cofactor for all Ala‐substituted Lys214, Glu306, and Glu515 residues of the active site network are independent of pH (with a slight increase at basic pH for Lys214Ala) except for Glu306Ala whose affinity constant shows a pK_a_ = 6.6 ± 0.4, similar to that of the WT (Figure 5). This suggests that the observed pK_a_ of ~6.5–7 of WT (also present in the log *k*_cat_ plot) is not attributable to Glu306. Thus, the Lys214‐Glu515 dyad could be the only suitable candidate responsible for the acidic pK_a_ of ~6.5–7.

Overall, a complex network at the enzyme active site is responsible for the protonation state of Cys340. The (second‐shell) Lys214‐Glu515 dyad appears to play two crucial roles: first, in the absence of the substrate SSA, they keep Cys340 thiolate prone to covalently bind NAD^+^ as a protective mechanism against hyperoxidation of hSSADH. This represents a new additional protective mechanism to the one based on the formation of a disulfide bond with Cys342 reported by Kim et al. (2009). Glu306 does not play any substantial role in this latter control mechanism. Second, in the presence of SSA, the networked dyad provides Glu306 with the correct protonation state to abstract/donate protons to Cys340, thus enhancing catalysis. Any factors altering these connections are predicted to impact hSSADH activity. It follows that SSADH deficiency pathogenic variants that affect, directly or indirectly, these network elements would cause loss‐of‐function.

## 3 CONCLUSIONS

We showed that the thiolate state of the hSSADH catalytic Cys340, unlike other aldehyde dehydrogenases (Munoz‐Clares et al. 2017), is mainly controlled at physiological conditions by a network of coordinated bonds and is protected from oxidation by the formation of a reversible adduct with its cofactor. Since the concentration of NAD^+^ in cells is 0.2–0.5 mM (Canto et al. 2015), which is considerably higher than the *K*_*D*_ of this hSSADH‐NAD^+^ binary complex, it can be argued that hSSADH mainly exists NAD^+^‐bound. When SSA enters the active site, NAD^+^ is displaced from Cys340 which performs a nucleophilic attack on the substrate aldehyde that has an affinity higher (about 1 μM) than NAD^+^, as extrapolated by the *K*_*m*SSA_ value. The complex reaction mechanism and the non‐competitive partial inhibition by SSA are described here. Given the low micromolar range of the SSA inhibition constant, this can be of physiological significance.

A regulatory mechanism of catalysis as well as of NAD^+^ binding is played by a network of residues, among which Glu515 seems to play a pivotal role. Data suggest that the acidic pK_a_ in both kinetic and *K*_*D*_ plots can be attributable to the intricate network (involving Lys214 and Glu306) that sustains Glu515. Its correct protonation state is mandatory to maintain the catalytic Glu306 deprotonated at physiological pH, so that it can act on Cys340 to trigger catalysis. At the same time, it directly controls cofactor binding and thiolate protection even in the absence of Glu306. The in silico analysis further suggests that such a complex network may be specific to the ALDH5 family, to which hSSADH belongs, and can be a shared feature for the whole family.

This combinatory approach underlines that catalysis is finely tuned not only by the catalytic residues responsible for the chemistry of the reaction but also by a second shell of residues responsible for regulating the catalytic ones. Thus, the subtle interactions among first‐shell catalytic residues and second‐shell networked residues might play the main role in hSSADH catalysis and/or oxidative insult protection. These findings are also potentially crucial to finding the rationale of the molecular basis for the pathogenicity of several SSADH deficiency variants, directly or indirectly influencing the identified active site network. From this study, the pathogenic effect of some variants of residues involved in interface stabilization (Arg173, Gly176, and Gly533), NAD^+^ binding (Gly268) and catalysis (Asn335) (Tokatly Latzer et al. 2023c) can be interpreted by small local changes that trigger wider consequences in a sort of *domino* effect that ultimately affect the active site architecture. This widens the possibility of defining a precision therapy approach based on the knowledge of each structural determinant that governs the total or partial loss‐of‐function.

## 4 MATERIALS AND METHODS

### 4.1 Materials

SSA, SA, NAD^+^, NADH, dithiothreitol (DTT), thrombin, β‐mercaptoethanol (BME), isopropyl‐β‐d‐thiogalactopyranoside (IPTG), phenylmethylsulfonyl fluoride (PMSF), and SigmaFast inhibitor cocktail were purchased from Merck (Darmstadt, Germany). Anti‐SSADH and anti‐His‐tag antibodies were from Santa Cruz Biotechnology (Dallas, TX). All other chemicals were of the highest purity available.

### 4.2 Determination of *K*_*D*_ for NAD ^+^

The equilibrium dissociation binding constant, *K*_*D*_, for NAD^+^ was determined by following the change in the intrinsic fluorescence (*λ*_exc_ = 295 nm, *λ*_em_ = 335 nm) of 0.1 μM enzymatic species (except for Glu306Ala whose concentration was set to 0.4 μM), measured by increasing NAD^+^ concentrations (0.1–100 μM). The changes in fluorescence quenching were plotted versus NAD^+^ concentrations and the data were fitted to a hyperbola equation to obtain the *K*_*D*_ value.

### 4.3 Determination of the steady state kinetic parameters, of the reaction mechanism and active site titration

Steady state kinetic parameters have been obtained by incubating 8 nM SSADH with increasing concentrations of SSA (from 1 to 200 μM) and of NAD^+^ (from 1 to 1500 μM) in 100 mM potassium phosphate buffer, pH 8 at 25°C. Initial rates were determined by following spectrophotometrically the signal of increase of NADH at 340 nm (*ε*_*M*_ = 6.22 mM^−1^ cm^−1^). The same assay was carried out at 14°C in the presence of 2 nM SSADH and different concentrations of the cosubstrates (0.2–500 μM SSA and 5–500 μM NAD^+^), by evaluating the concentration of NADH produced by exciting at 340 nm and measuring the change in fluorescence intensity at 445 nm. A calibration curve has been built correlating the change in fluorescence intensity to the concentration of NADH.

The obtained plots were fitted to the Michaelis–Menten equation (Equation (1)) to determine the apparent value of the kinetic parameters for both cosubstrates at subinhibitory SSA concentrations (1–10 μM), or to the Michaelis–Menten equation corrected for substrate inhibition when SSA was assayed at different concentrations (Equation (2)), where *K*_*m*_ represents the apparent affinity constant for SSA and *K*_*i*_ the apparent SSA inhibitory constant.

(1) $$V_{0}=\frac{{\mathrm{app}V}_{\max}∙\left[S\right]}{{\mathrm{app}K}_{m}+\left[S\right]},$$

(2) $$V_{0}=\frac{{\mathrm{app}V}_{\max}∙\left[S\right]}{{\mathrm{app}K}_{m}+\left[S\right]\;\left(1+\frac{\left[S\right]}{{\mathrm{app}K}_{i}}\right)}.$$

Global fitting of all data collected at subinhibitory SSA concentrations and full‐range NAD^+^ concentrations were then fitted to Equation (3) which accounts for an ordered bi–bi mechanism,

(3) $$V_{0}=\frac{V_{\max}\;\left[A\right]\left[B\right]}{\left(K_{\mathrm{iA}}K_{B}+K_{B}\left[A\right]+K_{A}\left[B\right]+\left[A\right]\left[B\right]\right)},$$

where *A* is the first substrate to bind to the enzyme, *B* is the second substrate and the one that exhibits substrate inhibition, *K*_*A*_ and *K*_*B*_ are the Michaelis constants for substrate *A* and *B*, respectively, and *K*_*iA*_ is the dissociation constant of *A* from its complex with the enzyme.

In the presence of inhibitory SSA concentrations (from 12 to 200 μM), data were fitted to Equation (4),

(4) $$V_{0}=\frac{V_{\max}\left[A\right]\left(1+\frac{\left[B\right]}{K_{\mathrm{ix}}}\right)}{K_{A}\left(1+\frac{\left[B\right]}{K_{\text{is}}}\right)+\left[A\right]\left(1+\frac{\left[B\right]}{K_{\mathrm{ii}}}\right)},$$

where *A* is the first substrate to bind to the enzyme, *B* is the second substrate and the one that exhibits substrate inhibition, *K*_*A*_ is the Michaelis constants for substrate *A*, *K*_*is*_ is the slope inhibition constant and *K*_*ix*_ and *K*_*ii*_ are intercepts inhibition constants.

Equation (5) was used for global fitting at full range concentrations of cosubstrates for a partial non‐competitive substrate inhibition simulation,

(5) $$V_{0}=\frac{V_{\max}\left[A\right]\left[B\right]\left(1+\frac{b\left[B\right]}{K_{\text{is}}}\right)}{\left(K_{\mathrm{iA}}K_{B}+K_{B}\left[A\right]+K_{A}\left[B\right]+\left[A\right]\left[B\right]\left(1+\frac{\left[B\right]}{K_{\text{is}}}\right)\right)},$$

where *A* is the first substrate to bind to the enzyme, *B* is the second substrate and the one that exhibits substrate inhibition, *K*_*A*_ and *K*_*B*_ are the Michaelis constants for substrates *A* and *B*, respectively, *b* is the factor that describes the effect of substrate inhibition on *V*_max_, *K*_*is*_ is the substrate inhibition constant affecting the slope and *K*_*iA*_ is the dissociation constant of *A* from its complex with the enzyme. With this fitting, the estimate of the latter parameter (*K*_*iA*_ is *K*_*D*NAD+_) is rough, while it was better measured by spectroscopic analyses (see above).

Product inhibition studies have been carried out by using different concentrations of NADH (from 17 to 220 μM) keeping SSA saturating (10 μM) at variable NAD^+^ concentrations. The obtained plots were fitted to linear regression curves that converged on the y‐axis, indicative of a competitive inhibition. Values of slopes and intercepts were plotted for *K*_*i*_ determination. Inhibition by 55 μM NADH is fitted to a competitive or non‐competitive model at the different combinations (subsaturating or saturating) of cosubstrate concentrations.

Active site titration was performed by adding different enzyme concentrations (4–14 μM) to a mixture containing 20 μM SSA and 500 μM NAD^+^. The absorbance changes at 340 nm were monitored at 4°C in 100 mM potassium phosphate buffer, 10 mM BME at pH 8 with a TC10‐100 (path length of 1 cm) quartz cell coupled to a BioKine PMS‐60 instrument using a stopped‐flow Biologic SFM300 spectrophotometer.

### 4.4 pH dependence of *K*_*D*NAD_ _+_ and of the kinetic parameters

The determination of the *K*_*D*_ for NAD^+^ at various pH values was carried out in 50 mM BTP, 10 mM BME at the desired pH (range 6–10). In details, 0.1 μM hSSADH was incubated with different NAD^+^ concentrations (0.1–100 μM) at each pH value and the related *K*_*D*_ was obtained as reported above. An appropriate blank was run in all experimental conditions and subtracted from samples. Results were fitted to Equation (6) and reported in logarithmic scale to obtain the pK_a_,

(6) $$K_{D}=C\left(1+10^{pK_{a}-\mathrm{pH}}\right),$$

where *C* represents the pH‐independent value of *K*_*D*_.

Kinetic parameters at different pH values were determined by incubating hSSADH (8 nM) with increasing concentrations of SSA (from 1 to 200 μM) and of NAD^+^ (from 1 to 1000 μM) in 50 mM BTP, 10 mM BME, in the pH range 5.5–10, at 25°C. Initial rates were determined by the increase of NADH concentration, followed spectrophotometrically at 340 nm (*ε*_*M*_ = 6.22 mM^−1^ cm^−1^). The values of *k*_cat_ and *k*_cat_/*K*_*m*_ were determined with Equations (1) or (2) and plotted versus pH. Kinetic parameters were then fitted to Equation (7) or Equation (8) to obtain the relative pK_a_ values,

(7) $$\mathrm{Log}\left(k_{\mathrm{cat}}\right)=\mathrm{Log}\left(\frac{C}{1+10^{pK_{a1}-\mathrm{pH}}}\right),$$

(8) $$\mathrm{Log}\left(\frac{k_{\mathrm{cat}}}{K_{m}}\right)=\mathrm{Log}\left(\frac{C}{1+10^{pK_{a1}-\mathrm{pH}}+1+10^{\mathrm{pH}-pK_{a2}}}\right),$$

where *C* is the maximal value observed for the referred parameter.

### 4.5 Statistical analysis

Statistical analysis was performed using the GraphPad Prism software (v. 5.02, La Jolla, CA) and OriginPro (v.10.1.0.170, OriginLab, Northampton, MA). The data are presented as mean values ± SD unless otherwise stated.

### 4.6 Sequence similarity networks

ALDH superfamily was represented by sequence similarity networks (SSNs) around the protein sequences coded by the 19 human ALDH genes. Each human ALDH protein (listed in Table 1) was used as query to search other homologous ALDH sequences from the UniRef100 database (03/2021 update), by employing three MMseq2 (release 13–45,111) search iterations (Mirdita et al. 2019; Steinegger and Soding 2017). The sequences collected from each query search were filtered by HHfilter 3.3.0 (Steinegger et al. 2019) to allow max 60% pairwise identity and at least 75% alignment coverage to each human ALDH query. The final list consisted of 11,571 ALDH homologous centroid sequences, for which Metadata annotations (i.e., taxonomy) were retrieved from NCBI taxonomy database (Federhen 2012) by *E‐Utilities* (Sayers 2022). This list of sequences was merged with other 21 proteins for which a biochemical record validating their SSADH activity was available in literature (Table 1). The final list of sequences (11,592) was pairwise aligned by Needleman‐Wunsch algorithm, as implemented in the EMBOSS software suite version 6.6.0 (Rice et al. 2000), using the BLOSUM62 scoring matrix with 10 and 0.5 as gap opening and gap extension penalties, respectively. ALDH SSNs were obtained in GraphML format by NetworkX version 1.9 (Hagberg et al. 2008): each sequence is a node of the network, with edges weighted by % identity, with minimum being 40%, as previously proposed as ALDH inter‐family threshold (Vasiliou and Nebert 2005). Networks containing only human ALDH or biochemically validated SSADH sequences (3487) were visualized in Cytoscape 3.9.1 (Su et al. 2014) using the Edge‐Weighted Spring Embedded Layout (Orlando et al. 2021), which places highly interconnected and similar sequences closer in bidimensional space.

### 4.7 Phylogenetic analysis

Molecular evolution of SSADHs in the context of ALDH superfamily was studied by estimating a Maximum Likelihood (ML) phylogenetic tree from an alignment of human ALDHs (19) and characterized SSADHs (16) sequences. PhyML 3.3.20211231 (Guindon et al. 2010) was used for 10 ML inference starting from random trees, with LG substitution model (Guindon et al. 2010) and a ML estimate of gamma distribution of site variation (four categories). Bootstrap values at nodes were added by using the gradual transfer distance method from 1000 bootstrap replicates (Lemoine et al. 2018). To generate the input alignment, mTM‐align (Version 20220104; Dong et al. 2018) was used to make a structure‐based multiple sequence alignment, using as input the monomeric 3D structures available from PDB and AlphaFold2 pre‐computed AF models from AlphaFold‐EBI (accessed at 11/11/2023; Varadi et al. 2024), while the structure was predicted for SSADHs without a PDB record and not in AlphaFold‐EBI, starting from the raw Uniprot primary sequence. Colabfold v.1.3.0 Jupyter Notebook AlphaFold2_mmseqs2 (https://github.com/sokrypton/ColabFold; Mirdita et al. 2022) was used with default parameters and a final energy minimization step; the structure model with the highest average predicted lDDT score was selected, and residues with predicted lDDT < 85 were trimmed. Sites with gaps in more than half of the sequences were removed before proceeding with the phylogenetic inference.

### 4.8 Direct coupling analysis of SSADH families

An SSADH family was defined by inspecting the distribution of characterized SSADHs in SSNs and the phylogenetic tree. A more detailed analysis was performed for each identified SSADH family. Characterized SSADHs from each SSADH family were clustered at 40% identity by CD‐HIT 4.8.1 to retrieve a per‐family centroid (Fu et al. 2012). HHblits 3.3.0 (Steinegger et al. 2019) was used to build an alignment of homologous sequences with at least 45% identity and 90% coverage to the centroid sequence of each SSADH family. UniRef100 (http://wwwuser.gwdg.de/~compbiol/uniclust/2021_03/) and BFD databases (https://bfd.mmseqs.com/) were employed. To avoid redundancy, the limit of max 90% pairwise identity was allowed. Metadata annotations (i.e., taxonomy) for non‐metagenomic sequences were retrieved from NCBI taxonomy database (Federhen 2012) by *E‐Utilities* (Sayers 2022). The family‐specific alignment was used to investigate the relative frequency of residues at sites important for catalysis and substrate binding in hSSADH, and to infer coevolution signals for residue pairs predicted to interact in hSSADH oligomerization at dimeric and tetrameric interfaces. Coupling scores between any residue pair were estimated by performing a direct coupling analysis with *plmc* (https://github.com/debbiemarkslab/plmc; Federhen 2012) on the alignment of each SSADH family. Three analyses were performed with different values for the *θ* parameter (0, 0.2, 0.3), which defines the minimum relative pairwise sequence difference that is used to down weight similar sequences. The per‐residue coupling scores for each alignment residue were normalized and averaged over replicates with different *θ* parameters. Only scores for residues distant >4 amino acids in the primary sequence were considered in the analysis, to remove any strong co‐evolutionary signal due to locally conserved functional patterns or secondary structure elements within the same chain.

### 4.9 Molecular dynamics simulations of tetrameric reduced hSSADH without NAD ^+^

Five independent molecular dynamics (MD) simulation replicates were performed with GROMACS 2019.6 (Abraham et al. 2015) using the AMBER14SB forcefield (Maier et al. 2015), as follows: the protonation states of *Homo sapiens* SSADH (PDB ID: 2W8O) were determined with respect to the dominant protonation state at pH 7.0 according to the predicted pKa (see above). The system was solvated in a dodecahedron box containing TIP3P parameterized explicit water molecules (Jorgensen et al. 1983). Na^+^ and Cl^−^ ions at 100 mM concentration were added to neutralize the negative charge of the system. Each system was energy minimized and equilibrated for 4 ns in NPT conditions at 298.15 K (25°C) (V‐rescale thermostat) and a constant pressure of 1 atm (Berendsen barostat). During equilibration, the positions of heavy atoms were restrained with a force constant of 1000 kJ mol^−1^ nm^−1^. Forty nanosecond productive MD simulations were performed for each equilibrated system (integration step of 2 fs, Nose‐Hoover thermostat, and Parrinello‐Rahman barostat), saving information every 0.1 ns. Three MD replicates have converged in the first 20 ns and we retained the frames after 20 ns for further analyses.

PyContact (https://github.com/maxscheurer/pycontact; Scheurer et al. 2018) was used to detect non‐covalent interactions that participate in hSSADH oligomerization, using only interactions at a max distance of 3.6 Å and present in at least 50% of analyzed simulation frames.

### 4.10 Flexible induced‐fit docking of reduced apo hSSADH

The PDB atomic coordinates of reduced hSSADH (PDB ID: 2W8O) were docked with NAD^+^ (C1=CC(=C[N+](=C1)C2C(C(C(O2)COP(=O)(O)OP(=O)(O)OCC3C(C(C(O3)N4C=NC5=C(N=CN=C54)N)O)O)O)O)C(=O)N) or SSA (O=CCCC(=O)O). The end‐to‐end deep generative method DynamicBind v.1.0 (https://doi.org/10.21203/rs.3.rs-3225151/v1) was used, to allow for full flexibility in the protein and ligand during the docking procedure, which allowed to obtain an induced‐fit complex. The top‐1 ranked solution for each docking attempt was retained.

## AUTHOR CONTRIBUTIONS

**Samuele Cesaro:** Conceptualization; investigation; formal analysis; methodology. **Marco Orlando:** Conceptualization; investigation; writing – original draft; methodology; formal analysis. **Ilaria Bettin:** Investigation; methodology; formal analysis. **Carmen Longo:** Investigation; methodology; formal analysis. **Giulia Spagnoli:** Investigation; methodology. **Patrizia Polverino de Laureto:** Investigation; methodology; formal analysis. **Gianluca Molla:** Conceptualization; validation; formal analysis; data curation; supervision. **Mariarita Bertoldi:** Conceptualization; funding acquisition; writing – original draft; validation; writing – review and editing; supervision; resources; data curation.

## CONFLICT OF INTEREST STATEMENT

The authors declare no conflicts of interest.

## Supporting information


**Figure S1.** Supporting Information.

**Data S1.** Supporting Information.
