# Supplementary material for: A crucial active site network of titratable residues guides catalysis and NAD + binding in human succinic semialdehyde dehydrogenase
Source: Protein Sci. 2024 Dec 28;34(1):e70024. doi: 10.1002/pro.70024 (PMC11681614; doi:10.1002/pro.70024)
Supplement: Supplementary file 1 — Figure S1. Supporting Information. [file PRO-34-e70024-s002.pptx]

## Slide 1
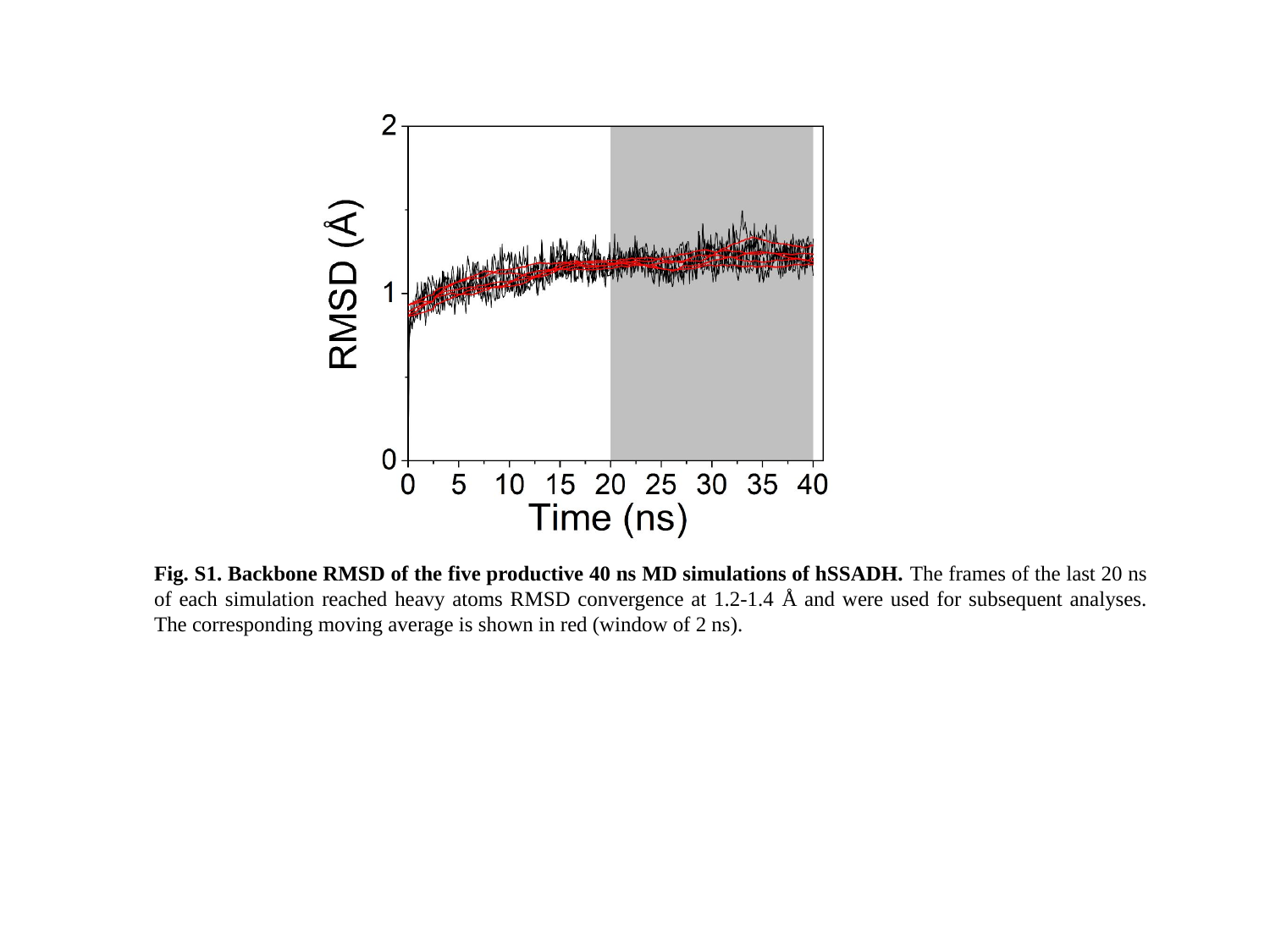

Fig. S1. Backbone RMSD of the five productive 40 ns MD simulations of hSSADH. The frames of the last 20 ns of each simulation reached heavy atoms RMSD convergence at 1.2-1.4 Å and were used for subsequent analyses. The corresponding moving average is shown in red (window of 2 ns).

## Slide 2
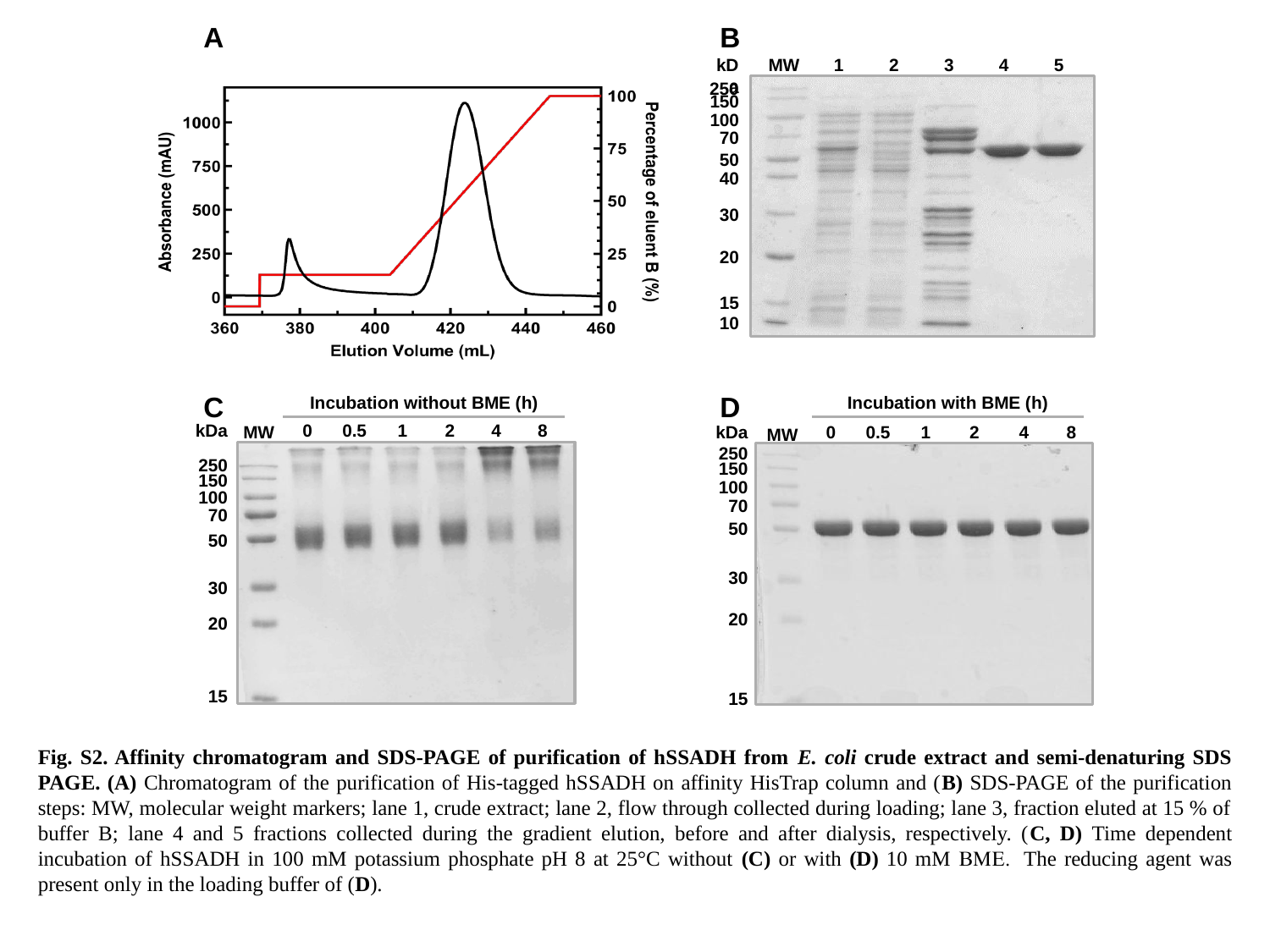

A
B
kDa
MW
1
2
3
4
5
250
150
100
70
50
40
30
20
15
10
Incubation without BME (h)
kDa
0
0.5
1
2
4
8
MW
250
150
100
70
50
30
20
15
Incubation with BME (h)
kDa
0
0.5
1
2
4
8
MW
250
150
100
70
50
30
20
15
C
D
Fig. S2. Affinity chromatogram and SDS-PAGE of purification of hSSADH from E. coli crude extract and semi-denaturing SDS PAGE. (A) Chromatogram of the purification of His-tagged hSSADH on affinity HisTrap column and (B) SDS-PAGE of the purification steps: MW, molecular weight markers; lane 1, crude extract; lane 2, flow through collected during loading; lane 3, fraction eluted at 15 % of buffer B; lane 4 and 5 fractions collected during the gradient elution, before and after dialysis, respectively. (C, D) Time dependent incubation of hSSADH in 100 mM potassium phosphate pH 8 at 25°C without (C) or with (D) 10 mM BME. The reducing agent was present only in the loading buffer of (D).

## Slide 3
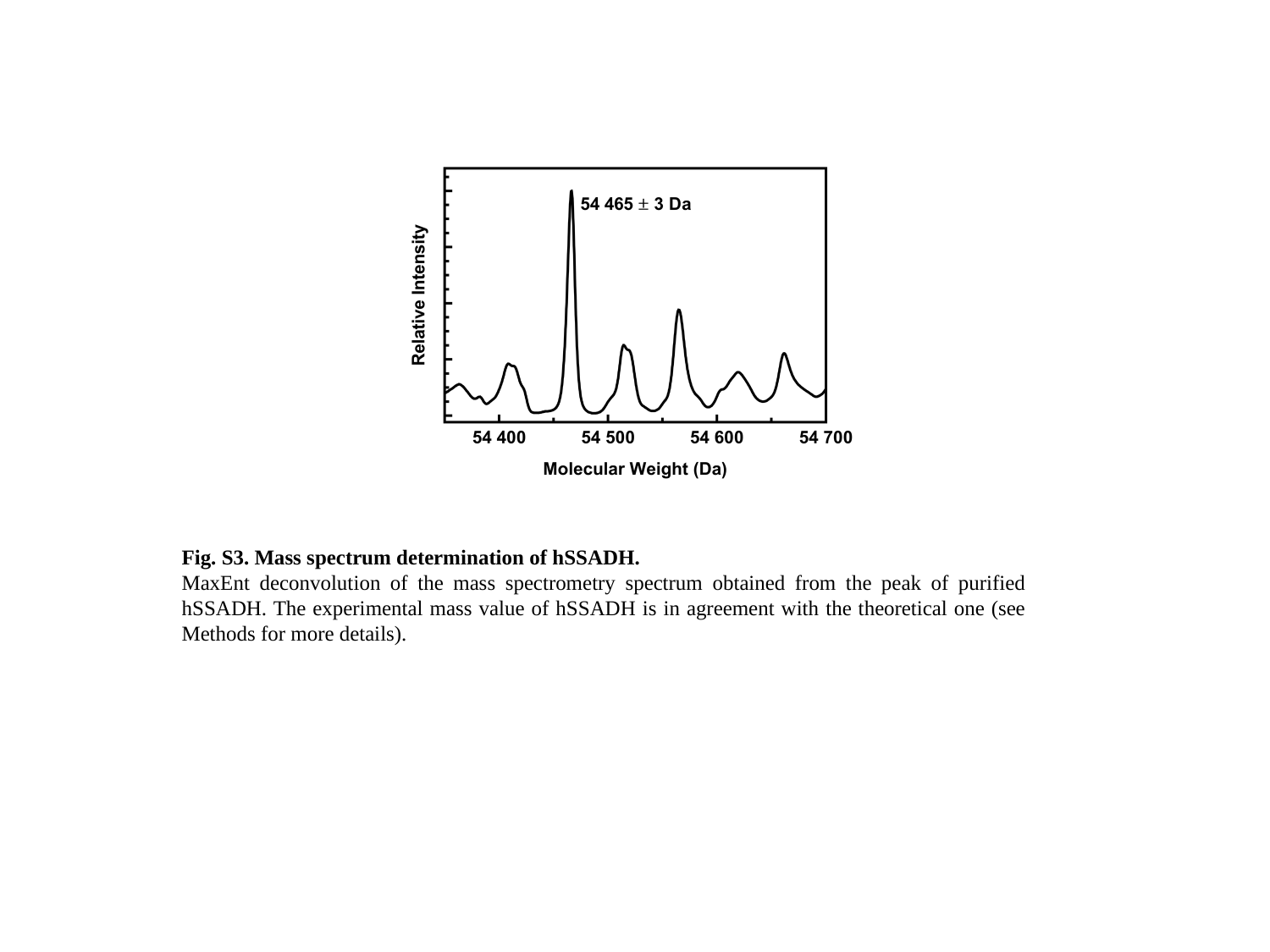

Fig. S3. Mass spectrum determination of hSSADH.
MaxEnt deconvolution of the mass spectrometry spectrum obtained from the peak of purified hSSADH. The experimental mass value of hSSADH is in agreement with the theoretical one (see Methods for more details).

## Slide 4
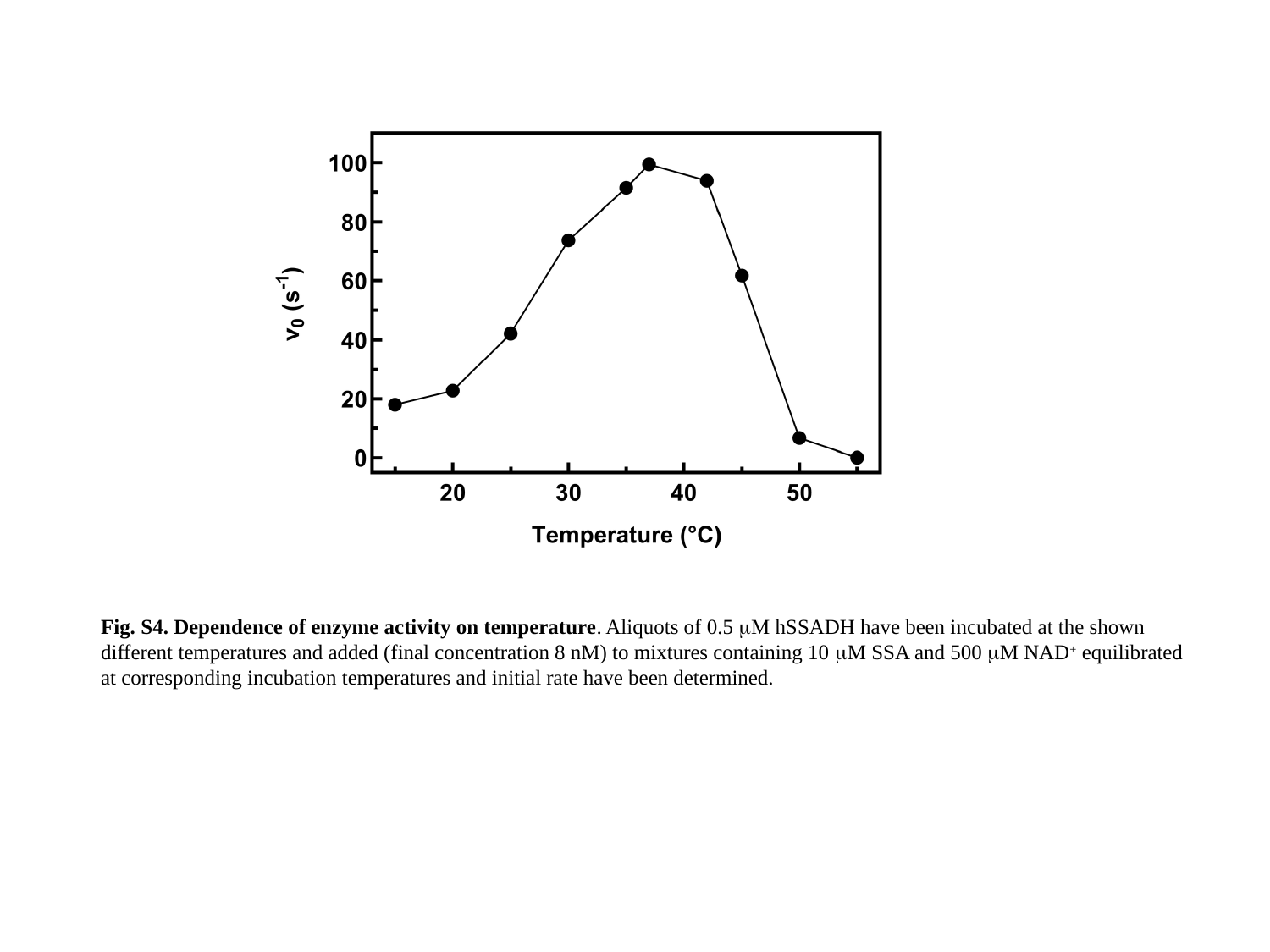

Fig. S4. Dependence of enzyme activity on temperature. Aliquots of 0.5 mM hSSADH have been incubated at the shown different temperatures and added (final concentration 8 nM) to mixtures containing 10 mM SSA and 500 mM NAD+ equilibrated at corresponding incubation temperatures and initial rate have been determined.

## Slide 5
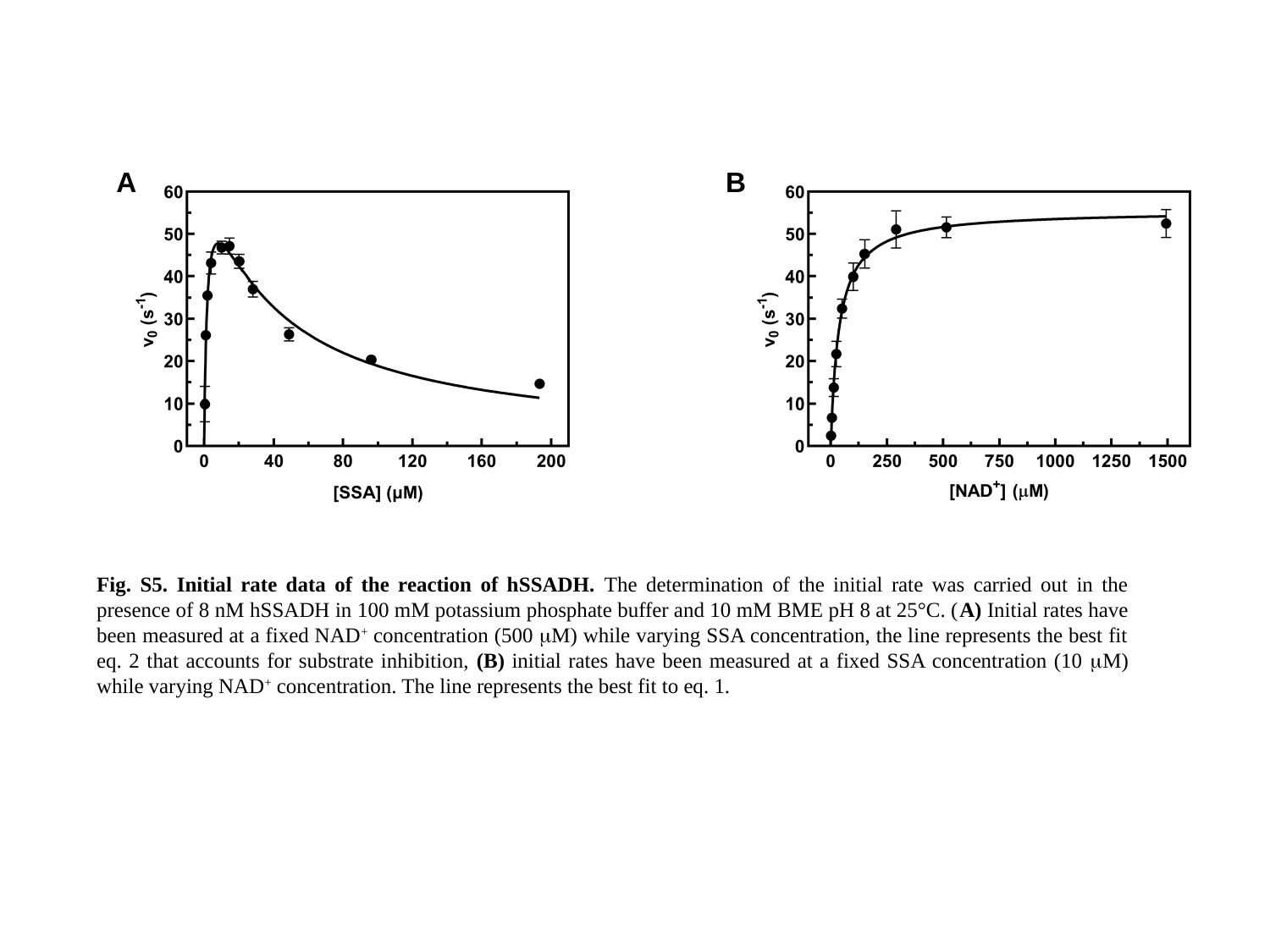

A
B
Fig. S5. Initial rate data of the reaction of hSSADH. The determination of the initial rate was carried out in the presence of 8 nM hSSADH in 100 mM potassium phosphate buffer and 10 mM BME pH 8 at 25°C. (A) Initial rates have been measured at a fixed NAD+ concentration (500 mM) while varying SSA concentration, the line represents the best fit eq. 2 that accounts for substrate inhibition, (B) initial rates have been measured at a fixed SSA concentration (10 mM) while varying NAD+ concentration. The line represents the best fit to eq. 1.

## Slide 6
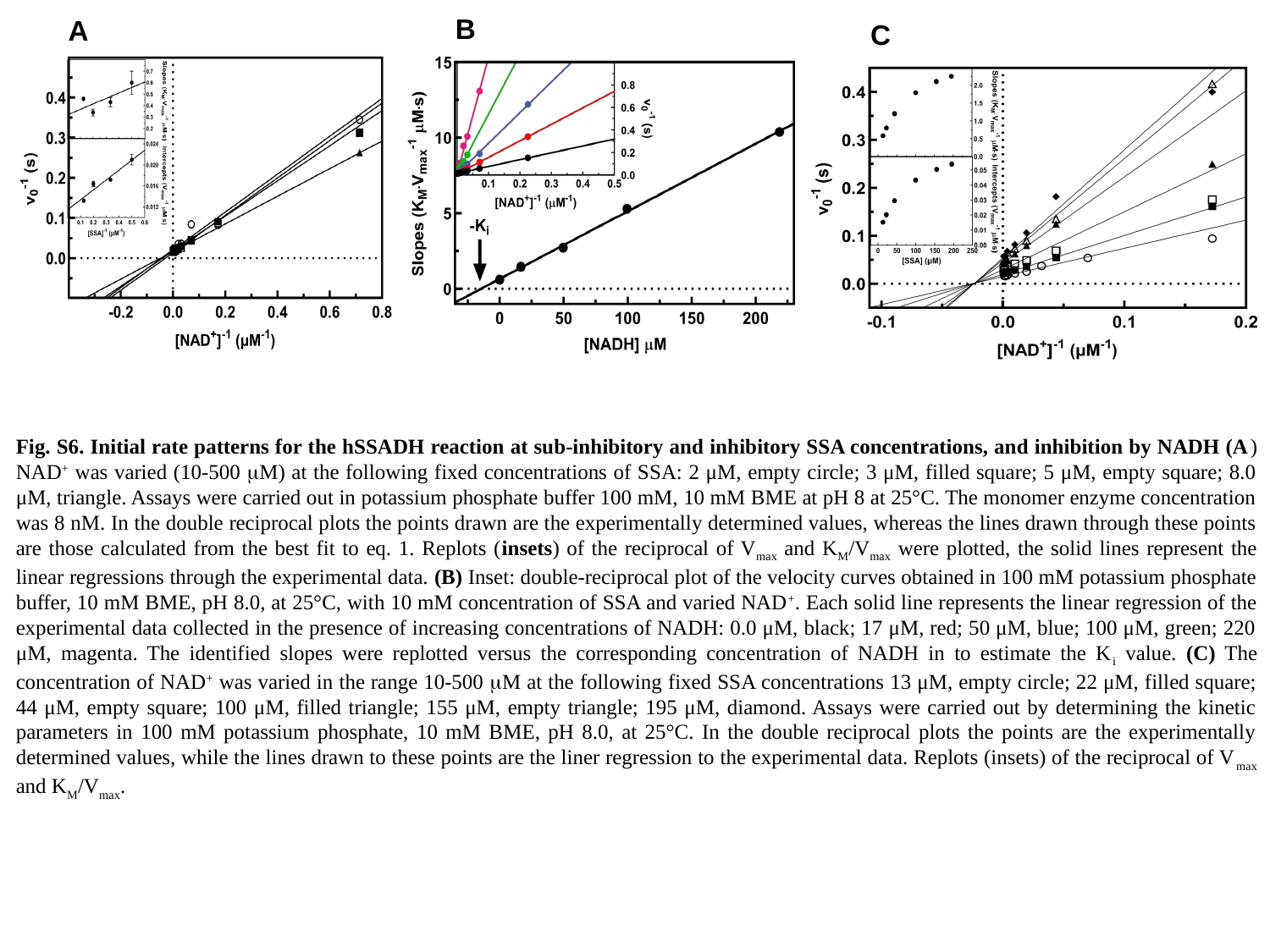

B
A
C
Fig. S6. Initial rate patterns for the hSSADH reaction at sub-inhibitory and inhibitory SSA concentrations, and inhibition by NADH (A) NAD+ was varied (10-500 mM) at the following fixed concentrations of SSA: 2 μM, empty circle; 3 μM, filled square; 5 μM, empty square; 8.0 μM, triangle. Assays were carried out in potassium phosphate buffer 100 mM, 10 mM BME at pH 8 at 25°C. The monomer enzyme concentration was 8 nM. In the double reciprocal plots the points drawn are the experimentally determined values, whereas the lines drawn through these points are those calculated from the best fit to eq. 1. Replots (insets) of the reciprocal of Vmax and KM/Vmax were plotted, the solid lines represent the linear regressions through the experimental data. (B) Inset: double-reciprocal plot of the velocity curves obtained in 100 mM potassium phosphate buffer, 10 mM BME, pH 8.0, at 25°C, with 10 mM concentration of SSA and varied NAD+. Each solid line represents the linear regression of the experimental data collected in the presence of increasing concentrations of NADH: 0.0 μM, black; 17 μM, red; 50 μM, blue; 100 μM, green; 220 μM, magenta. The identified slopes were replotted versus the corresponding concentration of NADH in to estimate the Ki value. (C) The concentration of NAD+ was varied in the range 10-500 mM at the following fixed SSA concentrations 13 μM, empty circle; 22 μM, filled square; 44 μM, empty square; 100 μM, filled triangle; 155 μM, empty triangle; 195 μM, diamond. Assays were carried out by determining the kinetic parameters in 100 mM potassium phosphate, 10 mM BME, pH 8.0, at 25°C. In the double reciprocal plots the points are the experimentally determined values, while the lines drawn to these points are the liner regression to the experimental data. Replots (insets) of the reciprocal of Vmax and KM/Vmax.

## Slide 7
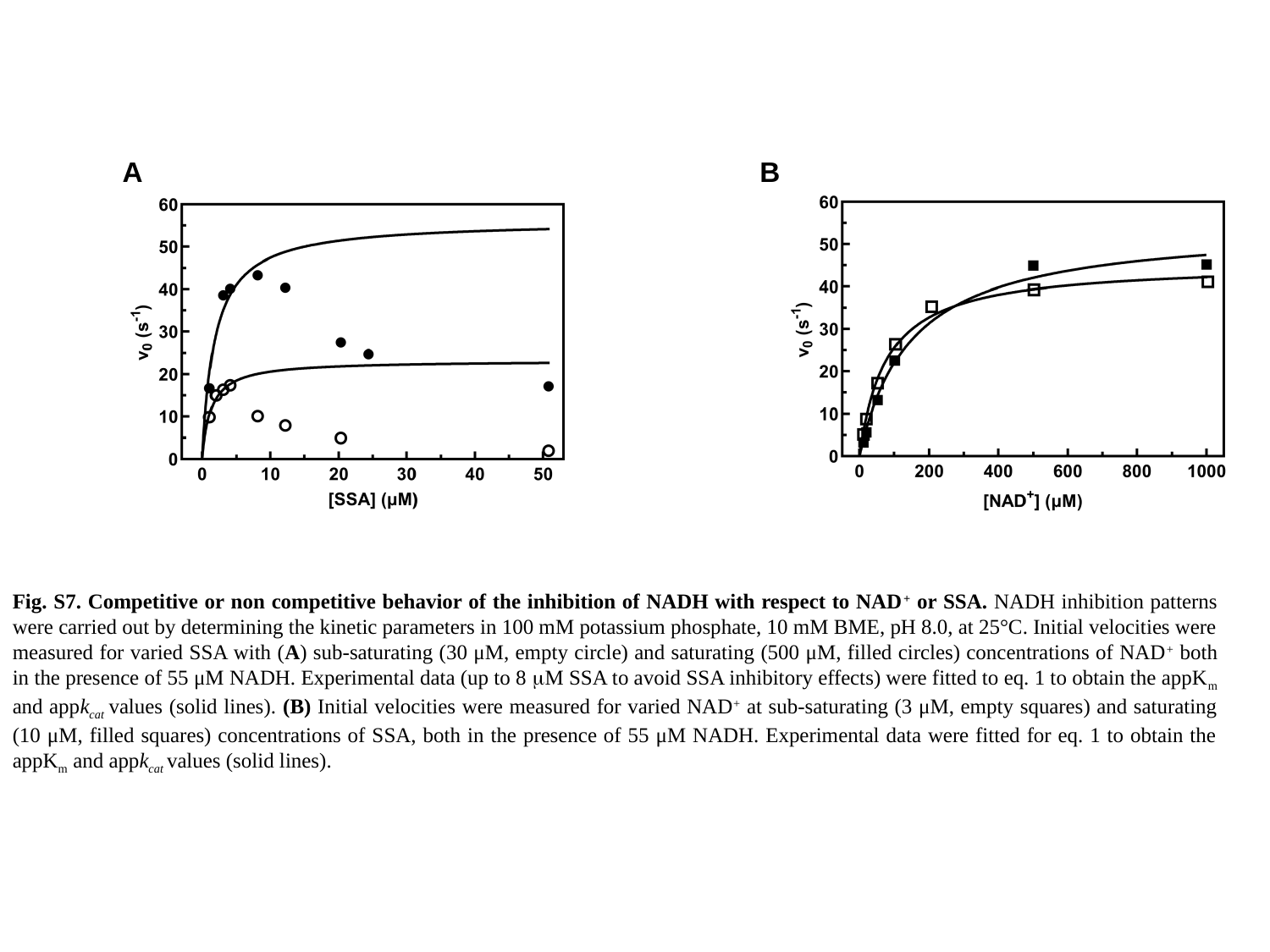

A
B
Fig. S7. Competitive or non competitive behavior of the inhibition of NADH with respect to NAD+ or SSA. NADH inhibition patterns were carried out by determining the kinetic parameters in 100 mM potassium phosphate, 10 mM BME, pH 8.0, at 25°C. Initial velocities were measured for varied SSA with (A) sub-saturating (30 μM, empty circle) and saturating (500 μM, filled circles) concentrations of NAD+ both in the presence of 55 μM NADH. Experimental data (up to 8 mM SSA to avoid SSA inhibitory effects) were fitted to eq. 1 to obtain the appKm and appkcat values (solid lines). (B) Initial velocities were measured for varied NAD+ at sub-saturating (3 μM, empty squares) and saturating (10 μM, filled squares) concentrations of SSA, both in the presence of 55 μM NADH. Experimental data were fitted for eq. 1 to obtain the appKm and appkcat values (solid lines).

## Slide 8
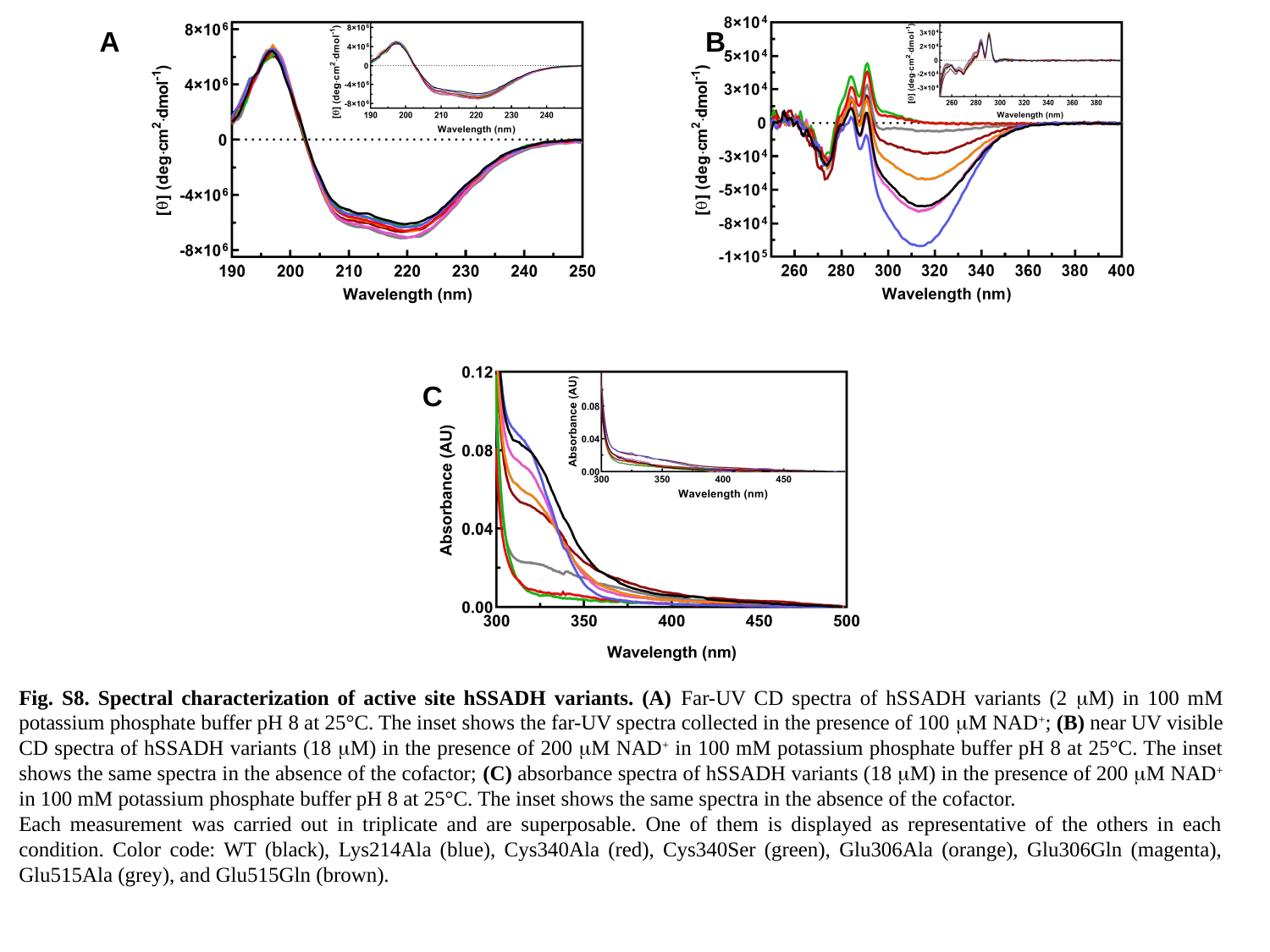

A
B
C
Fig. S8. Spectral characterization of active site hSSADH variants. (A) Far-UV CD spectra of hSSADH variants (2 mM) in 100 mM potassium phosphate buffer pH 8 at 25°C. The inset shows the far-UV spectra collected in the presence of 100 mM NAD+; (B) near UV visible CD spectra of hSSADH variants (18 mM) in the presence of 200 mM NAD+ in 100 mM potassium phosphate buffer pH 8 at 25°C. The inset shows the same spectra in the absence of the cofactor; (C) absorbance spectra of hSSADH variants (18 mM) in the presence of 200 mM NAD+ in 100 mM potassium phosphate buffer pH 8 at 25°C. The inset shows the same spectra in the absence of the cofactor.
Each measurement was carried out in triplicate and are superposable. One of them is displayed as representative of the others in each condition. Color code: WT (black), Lys214Ala (blue), Cys340Ala (red), Cys340Ser (green), Glu306Ala (orange), Glu306Gln (magenta), Glu515Ala (grey), and Glu515Gln (brown).
