# Supplementary material for: A crucial active site network of titratable residues guides catalysis and NAD + binding in human succinic semialdehyde dehydrogenase
Source: Protein Sci. 2024 Dec 28;34(1):e70024. doi: 10.1002/pro.70024 (PMC11681614; doi:10.1002/pro.70024)
Supplement: Supplementary file 2 — Data S1. Supporting Information. [file PRO-34-e70024-s001.docx]

**A crucial active site network of titratable residues controls catalysis and NAD^+^ binding in human succinic semialdehyde dehydrogenase**

Samuele Cesaro^1#^, Marco Orlando^2#§^, Ilaria Bettin^1^°, Carmen Longo^1^, Giulia Spagnoli^1^, Patrizia Polverino de Laureto^3^, Gianluca Molla^2^ and Mariarita Bertoldi^1^*

^1^Department of Neuroscience, Biomedicine and Movement Sciences, Section of Biochemistry, University of Verona, 37134 Verona, Italy

^2^Department of Biotechnology and Life Sciences, University of Insubria, 21100 Varese, Italy

^3^Department of Pharmaceutical and Pharmacological Sciences, University of Padova, 35131 Padova, Italy

^§^present institution: Department of Biotechnology e Biosciences, University of Milano-Bicocca, 20126 Milano, Italy

°present institution: Department of Biology, Friedrich-Alexander University, Erlangen-Nürnberg, Germany

#these authors contributed equally as co-first authors.

*To whom correspondence should be addressed: Mariarita Bertoldi

E-mail: mita.bertoldi@univr.it

**This PDF file includes:**

Supporting text: Supplemental Results and Supplemental Materials

Figures S1 to S8

Tables S1 to S7

SI References (18 references)

**Supplementary Information Text**

**Supplemental Results**

**WT hSSADH is highly expressed in bacteria and is more active under reducing conditions**

The enzyme purification procedure (**Fig. S2A**) leads to good yields (about 26 mg/L) of pure recombinant enzyme **(Table S2)**, as shown on SDS-PAGE (**Fig. S2B)**. The identity of the protein was assessed by mass spectrometry, giving a protein with a molecular weight of 54,465 ± 3 Da, compatible with the monomer of hSSADH (**Fig. S3**). The presence of a reducing agent such as 10 mM BME or 1 mM DTT prevents turbidity due to protein aggregation of possible exposed cysteine residues prone to covalently binding through disulfide bridges. By examining the spatial structure (PDB ID 2W8O), we mapped the position of the eleven cysteine residues present in each monomer. Five of them (Cys93, Cys110, Cys223, Cys229 and Cys272) map on the NAD^+^ binding domain, another five (Cys340, Cys342, Cys426, Cys434 and Cys502) on the catalytic domain and Cys531 on the oligomerization domain. Notably, Cys110, Cys426, Cys434, Cys502 and Cys531 are exposed to the surface of each monomer. No intrachain or interchain disulfide bonds are visible in the electron density map, corroborating that aggregation probably involves the exposed cysteine residues. By the way, the only visible disulfide bond is established in each monomer in the catalytic loop of the enzymatic oxidized form (PDB ID 2W8N) between Cys340 and Cys3 for regulatory control (Kim et al. 2009). A time-dependent conversion to high molecular weight species can be observed when WT hSSADH is incubated without a reducing agent while only one band is visible in the presence of a reductant (**Fig S2C,D**). Differently from (Kim et al. 2009), hSSADH is also active without a reducing agent, whereas its presence slightly enhances activity (+ 15%). The reason for this discrepancy is unknown; however, our results are identical to those collected on the human enzyme by Kang et al. (Kang et al. 2005), showing that SSADH seems to require a reducing agent to enhance stability, but it is not essential for catalysis.

We have already determined oligomeric size to be compatible with a homotetramer (Didiasova et al. 2024) from 0.1 to 5 mg/mL, confirming the results of (Kang et al. 2005). Dynamic Light Scattering (DLS) analyses (data not shown) show a hydrodynamic radius of 6.7 ± 0.6 nm, in line with a tetrameric assembly, taking into account that the gyration radius of the monomer of the three-dimensional structure is 2.38 nm and of the built tetramer is 3.57 nm (Kim et al. 2009). The optimal temperature was at 37°C (**Fig. S4)**.

The presence of the his-tag does not influence enzyme activity to a significant extent (data not shown). Thus, the his-tagged hSSADH was used in all the following experiments and denoted as hSSADH.

**Determination of the kinetic parameters of the reaction catalyzed by hSSADH and product inhibition studies**

Initial rates of the dehydrogenase reaction have been determined at increasing concentrations of SSA or NAD^+^ at fixed concentrations of the other co-substrate (**Fig. S5A,B**). We fitted the initial rate data in the presence of 10 μM SSA and variable NAD^+^ concentrations to eq. 1 (for all equations see Materials and Methods section of the main text) and obtained an apparent *k_cat_* = 68 ± 1 s^-1^, and an apparent K_mNAD+_ = 32 ± 2 μM. When SSA was the variable substrate at 500 μM NAD^+^, data were fitted to eq. 2 and the extrapolated apparent kinetic parameters were *k_cat_* = 124 ± 24 s^-1^, K_mSSA_ = 5 ± 2 μM, = and K_iSSA_ = 24 ± 8 μM. Notably, no lag times were revealed in the kinetic measurements, regardless of the addition order to the two co-substrates, nor their pre-incubation with the enzyme.

The obtained parameters suggest that substrate inhibition affects the determination of the actual values. However, we realized that SSA concentrations could be varied over a reasonable range before substrate inhibition occurs. Thus, initial rates versus different NAD^+^ concentrations (10-500 μM) in the range of subinhibitory SSA concentrations (1-10 μM) have been plotted **(Fig. S6A)** and fitted to eq.1. Similar results were obtained when NAD^+^ was the variable substrate. The double reciprocal plots were linear with standard intersection patterns at the left of the *y*-axis, suggesting either an ordered or a random bi-bi mechanism. Both the slope and the intercept replots (**Fig. S6A, insets**) were linear, yielding *k_cat_*= 80 ± 10 s^-1^, K_mSSA_ = 0.6 ± 0.2 μM and K_mNAD+_ = 14 ± 8 μM (**Table 2**). The global fitting with eq. 3 gave the following extrapolated values: *k_cat_*= 87 ± 4 s^-1^, K_mSSA_ = 1.5 ± 0.2 μM and K_mNAD+_ = 36 ± 5 μM. The latter can be considered as the best estimate of the kinetic parameters under these experimental conditions.

Product inhibition studies were performed to discriminate among ordered or random bi-bi mechanisms of hSSADH reaction. SA does not exert inhibitory effects up to 3 mM, suggesting that it is the first product to be released, a common feature among aldehyde dehydrogenases (Henehan and Tipton 1991; Munoz-Clares et al. 2010; Valenzuela-Soto and Munoz-Clares 1993; Vojtechova et al. 1997). At first, we determined that NADH is a competitive inhibitor of NAD^+^ (**Fig. S6B**) with a calculated competitive inhibition constant K_i_ value of 15 ± 2 μM, obtained by varying the concentrations of NAD^+^ at a constant subinhibitory concentration (10 μM) of SSA (**Fig. S6B inset**).

Thereafter, at variable sub-saturating SSA concentrations (up to 10 μM), NADH (55 μM, a concentration adequate to exert inhibition) is a non-competitive inhibitor at non-saturating NAD^+^ concentration, while this inhibition is alleviated at saturating NAD^+^ concentration (**Fig. S7, Table S3**).

NADH has been previously demonstrated to be a competitive or non-competitive inhibitor of the dehydrogenase reaction towards NAD^+^ or SSA, respectively, even if the reported inhibition constant values are higher (a value of about 100 μM for both constants) (Kang et al. 2005) than those obtained by our extensive kinetic analysis.

As reported in the literature (Munoz-Clares and Casanova-Figueroa 2019; Valenzuela-Soto and Munoz-Clares 1993; Vojtechova et al. 1997), fitting carried out only at sub-inhibitory aldehyde concentrations could lead to underestimates in V_max_ and K_m_ values. Thus, we also analyzed the kinetics of the reaction at higher inhibitory concentrations of SSA (12-200 μM) and at all SSA concentrations. In detail, we analyzed the kinetics of the reaction at higher inhibitory concentrations of SSA (12-200 μM) (**Fig. 3C**), while varying NAD^+^ concentrations (10-500 μM). We obtained a pattern of lines intersecting at the left of the *y*-axis, indicative of non-competitive inhibition. Replots of intercepts and slopes are curved in agreement with a partial inhibition (**Fig S6C insets**). Fitting to eq. 4 (Vojtechova et al. 1997) resulted in the inhibition constants reported in **Table 2.** At these inhibitory SSA concentrations, the estimate for *k_cat_* is even higher than at subinhibitory SSA concentrations. Thus, all data (at inhibitory and subinhibitory SSA concentrations) were then fitted to eq. 5 for a partial non-competitive substrate inhibition (Munoz-Clares and Casanova-Figueroa 2019), resulting in *k_cat_*= 166 ± 33 s^-1^, K_mSSA_ = 4 ± 1 μM and K_mNAD+_ = 84 ± 23 μM. K_iSSA_ 9 ± 3 μM, b = 0.03 ± 0.008.

Given the estimated high catalytic constant (about 160-170 s^-1^), in order to collect more accurate values of the affinity constants, especially at the low SSA concentrations, we decided to measure the initial velocity at 14 °C by fluorescence spectroscopy exciting at 340 nm by following NADH formation since by fluorescence we could increase the sensitivity (see Methods for details). Global fitting to all data with eq. 5 results in the following values for the kinetic parameters: *k_cat_*= 26 ± 2 s^-1^, K_mSSA_ = 1.2 ± 0.2 μM and K_mNAD+_ = 31 ± 5 μM, K_iSSA_ 13 ± 3 μM, b = 0.06 ± 0.01. While at this low temperature the catalytic constant is decreased, as expected, the affinity values are more readily evaluated and could represent the most reliable estimate of the co-substrates affinity values for hSSADH. The SSA inhibition constant is similar at both temperatures with a value of about 10-15 μM.

**Cya340Ala, Cys340Ser, Glu306Ala, Glu306Gln, Glu515Ala, Glu515Gln and Lys214Ala variants display spectroscopic features similar to those of the WT hSSADH**

All hSSADH variants can be purified with yields similar to the WT, except for Glu515Gln, which recovers at about 10% yields. The far UV circular dichroic (CD) spectra, either in the absence (**Fig. S8A**) or the presence of NAD^+^ (**Fig. S8A inset**), do not exhibit relevant differences, revealing no gross secondary structure modifications concerning the WT.

The thermal stability (**Table S6**) is comparable or slightly higher than the WT (**Table S4**) under the same experimental conditions and is not significantly increased (< 2°C) by the presence of NAD^+^, unless for Lys214Ala that behaves as the WT. The spectroscopic characterization shows that all variants, except for Cys340Ala and Cys340Ser, present a spectral “signature” of NAD^+^ binding, even if Glu515Ala responds with faint spectral modifications to all biophysical techniques applied. Near UV and visible CD spectra of the variants are unaltered concerning the WT (**Fig. S8B inset**), while the addition of 200 mM NAD^+^ determines different effects (**Fig. S8B**). Cys340 variants do not exhibit signals, suggesting that the cofactor does not bind to them. At the same time, Glu515Ala presents a weak negative broad signal, indicative of a marked modification of the cofactor binding environment or affinity. All the others show a negative band with a minimum at 315 nm, as the WT, but less intense, except for Lys214Ala. Absorbance spectra of all variants in the presence of NAD^+^ display a shoulder or a broad absorbance in the 320 nm region, except for the Cys340 variants, while Glu515Ala has a modest increment of signal in the same area (**Fig. S8C**). All the variants, such as the WT, do not show any signal in the visible region in the absence of NAD^+^ **(Fig. S8C inset)**.

**Supplementary Methods**

**Cloning, expression and purification of WT hSSADH**

The cDNA of WT hSSADH (isoform 2, NCBI Reference Sequence: NM_001080.3, the most active and abundant in cells) was a generous gift from Prof. Ritva Tikkanen (University of Gießen, Germany). It was initially inserted into pcDNA3 vector, excised with EcoRI and XhoI restriction enzymes, deprived of the mitochondrial peptide sequence (amino acids 1-47), optimized in GC content for expression in bacteria, cloned with NdeI and XhoI recognition sequences and inserted into pET15b, immediately after the his-tag and the thrombin sequence, to transform *E. coli* BL21 cells. WT hSSADH expression and purification was carried out as reported in (Didiasova et al. 2024). Protein monomer concentration was determined by using an ε_M_ = 49,390 M^-1^ cm^-1^ at 280 nm. Generally, enzyme concentration is expressed as monomeric. Instead, for activity assays the concentration is expressed as tetrameric, since the functionally active species is the tetramer.

The removal of the his-tag of hSSADH has been obtained by overnight incubation of his-tagged hSSADH with thrombin (1 unit for about 60 nanomoles of monomeric hSSADH) in 100 mM potassium phosphate buffer, 150 mM NaCl and 10 mM BME pH 8, at room temperature, followed by size exclusion chromatography with the same buffer at 25°C and Western Blot as described below.

Activity assays were performed in 100 mM potassium phosphate buffer and 10 mM BME pH 8 at 25°C by incubating 8 nM tetrameric WT enzyme with 10 μM SSA and 500 μM NAD^+^. The formation of NADH was followed spectroscopically at 340 nm (ε_M_ = 6,220 M^-1^ cm^-1^) or by fluorescence spectroscopy by exciting at 340 nm and following emission at 445 nm. In this latter assay, NADH formation was measured from a calibration curve prepared at known NADH concentrations (0.5-10 mM).

**Site-directed mutagenesis, expression, purification and characterization of variants**

All the variants have been obtained by Quick-Change II Mutagenesis Kit (Agilent Technologies, Santa Clara, California, USA) using the DNA oligos reported in **Table S7.** All mutations were confirmed by DNA sequence analysis of the whole ORF. Expression, purification and activity assay of the variants have been carried out in the same conditions of the WT protein, and the following enzyme concentrations were used: 0.25 μM for Cys340Ala and Cys340Ser, 0.5 μM for Glu306Ala and Glu306Gln, 0.1 μM for Glu515Ala, 0.02 μM for Glu515Gln and 0.35 μM for Lys214Ala.

**SDS polyacrylamide gel electrophoresis (PAGE) and immunoblotting**

SDS-PAGE was carried out with cast-in-place 12% polyacrylamide gels, which were loaded with samples prepared in Laemmli sample buffer and thermally treated for 5 min at 95 °C. After the electrophoretic run, which was conducted in 25 mM Tris, 192 mM glycine, 0.1% SDS, pH 8.3 buffer, at fixed voltage (i.e., 200 V) for 45 min, proteins were revealed by Coomassie staining or by immunoblotting. In the first case, the polyacrylamide gel was treated for 30 minutes at room temperature with a water solution containing 0.1% Coomassie Blue R250, 10% acetic acid, and 50% methanol. Then, to allow protein visualization, it was decolored with a water solution containing 10% acetic acid, and 40% ethanol. In the second case, the proteins were electrophoretically transferred onto a Immobilon-P, PVDF membrane (Merck Millipore, Burlington, Massachusetts, USA) in 25 mM Tris, 200 mM glycine, 0.1% Tween-20, 20% methanol, pH 8.5 buffer (TBST), at fixed voltage (i.e., 25 V per gel slab) for 1.5 h, with a wet apparatus. The membrane was blocked for 30 min with 5% (w/v) skim dry milk in TBST, and then hybridized with the primary antibody overnight at 4 °C. After three washing steps in TBST, the membrane was hybridized with the secondary antibody conjugated with horseradish peroxidase (HRP) for 1 h at room temperature. Both antibody incubating solutions were prepared in 5% (w/v) skim dry milk in TBST. ECL (Thermo Fisher Scientific, Inc., Waltham, Massachusetts, USA) was employed as substrate for the chemiluminescent reaction.

**Mass spectrometry (MS)**

WT hSSADH, at a final concentration of 0.2 mg/mL, was treated in 7M guanidine hydrochloride (GdnHCl) for 1 h. The resulting denatured protein was loaded in a Reverse Phase-High Performance Liquid Chromatography (RP-HPLC) system (1200 series Agilent Technologies, Santa Clara, California, USA), which was connected to a Jupiter C4 column (4.6 cm x 250 mm, Phenomenex, CA, USA). The mobile phases employed were a composition of two eluents: (A) 0.1% v/v of trifluoroacetic Acid (TFA) in water; (B) 0.085% (v/v) TFA in acetonitrile. These were combined, in a gradient elution, at a fixed flow of 1 mL/min. The peaks eluted from the column were collected and lyophilized in a vacuum concentrator (Savant SpeedVac., Thermo Fisher Scientific Inc.) and then analyzed by MS. For this purpose, a Xevo® G2-XS ESI-Q-TOF mass spectrometer (Waters Corporation, Milford, Massachusetts, USA), with a mass resolving power of about 40,000, has been employed for high spectra resolution of the recombinant hSSADH. The analysis was carried out in a 0.23% formic acid aqueous-acetonitrile 1:1 solution as mobile phase. All collected data were processed and analyzed by MassLynx. mass spectrometry software (Waters Corporation, Milford, Massachusetts, USA) employing the maximum entropy deconvolution (MaxEnt).

**Determination of the oligomeric size**

DLS experiments were carried out in a Nano Zetasizer instrument (Malvern, UK) with an enzymatic solution of 0.1 mg/mL in 100 mM potassium phosphate buffer at pH 8.0 at 25°C. Data were collected and analyzed with the appropriate manufacturer software.

**Differential scanning calorimetry (DSC)**

A concentration of 64 μM of SSADH, dissolved in 100 mM potassium phosphate buffer, 10 mM BME at pH 8.0, was analysed in a Nano DSC instrument (TA instruments, Waters Corporation, Milford, Massachusetts, USA) at 1°C/min in the range 25-90 °C. The experiment was carried out in the absence or presence of 200 μM NAD^+^ or 1 mM DTT. Data obtained were processed by the proprietary software NanoAnalyze.

**Activity and optimal temperature**

Residual activity at different temperatures (15-55 °C) was evaluated by determining the initial rate following 8 nM hSSADH incubation (previously equilibrated 20 min at 0.5 μM concentration at the corresponding temperature) in the presence of saturating concentrations of co-substrates (10 μM SSA and 500 μM NAD^+^), in 100 mM potassium phosphate buffer at pH 8.

**Spectroscopic analyses**

All spectroscopic measurements were carried out in 100 mM potassium phosphate buffer, pH 8 at 25°C unless otherwise stated. Absorbance spectra were recorded by using a Jasco V-550 spectrophotometer, and intrinsic fluorescence emission analyses (λ_exc_ = 295nm, λ_em_ = 335 nm) were carried out with a Jasco FP-8500 spectrofluorometer at 0.02 mg/mL hSSADH. Due to possible inner filter effects, NAD^+^ concentrations were increased up to 1000-fold excess with respect to SSADH concentration (ε_M_ NAD^+^ at 260 nm = 17,800 M^-1^ cm^-1^ (Pometun et al. 2022)). All reported spectra were corrected for dilution. The possible inner filter effect was minimized by using an enzyme concentration with an absorbance of less than 0.1 units at 280 nm and was subtracted by measuring the fluorescence intensity of 0.6 μM free tryptophan with 0-100 μM NAD^+^ as reported (Yammine et al. 2019). CD spectra were determined with a Jasco J-1500 spectropolarimeter at a scan speed of 50 nm/min with 2 nm bandwidth at 0.1 mg/mL and 1 mg/mL protein concentrations for the far UV (190-250 nm) and near UV-visible (250-400 nm) range, respectively. Thermal denaturation was performed by monitoring the CD signal at 222 nm at 0.1 mg/mL SSADH on a 25-90°C linear temperature gradient with a temperature slope of 1.5°C/min. Deconvolution of far UV CD spectra was carried out by BeStSel (Micsonai et al. 2022).

**pK_a_ titration state prediction**

The side chain pKa of ionizable residues exposed in the active site of hSSADH was predicted by averaging the predictions of propka v.3.5 (Olsson et al. 2011), pyPKA (Reis et al. 2020), pKAI^+^ (Reis et al. 2022), DeepPKA (Cai et al. 2021) and pKa-ANI (Gokcan and Isayev 2022) on the WT structure (PDB ID: 2W8O). Ensemble-based constant pH MD simulation approaches were not used as they were usually not shown to provide consistent improvements in cysteine pKa prediction (Awoonor-Williams et al. 2023), while there are general difficulties in reaching convergence of sidechain torsion angle/titration state sampling (Buslaev et al. 2022).

**Table S1. List of pairwise non-covalent interactions at a distance ≤ 3.6 Å between hSSADH protomers, observed in at least 50% of productive MD simulation frames.** The EC scores (Averaged over different θ parameters) between non-covalent interacting residue pairs for the two SSADH families are reported. As common practice, only the scores for residues distant > 5 amino acids in the primary sequence were considered. These scores were plotted in **Fig. 2B**. For further details about how they were calculated, see the Materials and Methods section. The homologous correspondence between alignment sites of the two families was retrieved by the structure-based alignment of reference sequences of each alignment (hSSADH with PDB ID: 2W8O for *ALDH5s* and StSSADH with PDB ID: 3ETF for *Other SSADHs*).

| **Residue 1** | **Residue 2** | **Type of interaction** | **Interface** | **Frequency**  **(%) of MD frames)** | **EC scores** | | | | |
| --- | --- | --- | --- | --- | --- | --- | --- | --- | --- |
|  |  |  |  |  | **ALDH5s ± SD** | | | **Other SSADHs**  **± SD** | |
| Tyr529 backbone | Val490 backbone | Van der Waals | Dimeric | 1 | -0.090324456 **±**  0.355940795 | | -0.4635 **±**  0.241952 | | |
| Leu527 backbone | Arg514 sidechain | hydrogen bond | Dimeric | 1 | 2.866242726 **±**  0.464398496 | | 1.934751 **±**  0.020698 | | |
| Cys531 backbone | Val492 backbone | hydrogen bond | Dimeric | 1 | 1.897495899 **±**  0.243678515 | | -0.41585 **±**  0.367328 | | |
| Val492 backbone | Gly533 backbone | hydrogen bond | Dimeric | 1 | 0.89669124 **±**  0.47562686 | | -0.29952 **±**  0.23146 | | |
| Arg173 sidechain | Glu170 sidechain | salt bridge | Tetrameric | 1 |  | |  | |  |
| Cys531 backbone | Val490 backbone | hydrophobic contact | Dimeric | 0.9975 | 1.33293325 **±**  0.910207608 | | 1.386467 **±**  0.527222 | | |
| Ile178 backbone | Gly176 backbone | Hydrogen bond | Tetrameric | 0.9975 |  | |  | |  |
| Arg173 sidechain | Glu169 sidechain | salt bridge | Tetrameric | 0.995 |  | |  | | |
| Tyr529 backbone | Gly488 backbone | hydrogen bond | Dimeric | 0.9925 | -0.132515445 **±**  0.298414668 | | -0.0985 **±**  0.114588 | | |
| Tyr519 sidechain | Asp177 sidechain | hydrogen bond | Dimeric | 0.985 | 27.7996679 **±**  2.350254699 | | 7.377039 **±**  0.112737 | | |
| Arg173 sidechain | Trp166 backbone | stacking | Tetrameric | 0.965 | 0.353640746 **±**  0.179664263 | | 0.684833 **±**  0.445858 | | |
| Lys518 sidechain | Asp177 sidechain | salt bridge | Dimeric | 0.905 | 14.2628896 **±**  0.977336761 | | 9.713511 **±**  0.802874 | | |
| Tyr529 sidechain | Ile497 backbone | hydrogen bond | Dimeric | 0.895 | 3.964212816 **±**  0.906369267 | | 0.356287 **±**  0.127492 | | |
| Tyr529 backbone | Met489 backbone | Van der Waals | Dimeric | 0.8525 | 0.780157692 **±**  0.847974086 | | 1.49781 **±**  0.9431 | | |
| Lys118 sidechain | Glu165 sidechain | salt bridge | Tetrameric | 0.805 | 6.731088234 **±**  0.714121319 | | -0.68553 **±**  0.74362 | | |
| Arg187 sidechain | Glu483 sidechain | salt bridge | Tetrameric | 0.8025 | 13.82039351 **±**  2.325039576 | | -0.14982 **±**  0.405874 | | |
| Ile178 backbone | Tyr175 backbone | hydrogen bond | Tetrameric | 0.7975 |  |  |  | |  |
| Arg186 sidechain | Leu496 backbone | hydrogen bond | Dimeric | 0.7925 | 2.575668097 **±**  0.380392582 | | 0.41213 **±**  0.218348 | | |
| Lys528 backbone | Gly488 backbone | Van der Waals | Dimeric | 0.7625 | -0.04616299 **±**  0.056470052 | | -0.05486 **±**  0.152559 | | |
| Arg172 sidechain | Glu501 sidechain | salt bridge | Tetrameric | 0.7525 | 6.424292466**±**  0.811860183 | | -0.40296 **±**  0.160252 | | |
| Tyr529 sidechain | Cys502 sidechain | Van der Waals | Dimeric | 0.7325 | 3.339065161 **±**  0.152907741 | | 6.201535 **±**  1.182339 | | |
| Pro475 sidechain | Gln 473 backbone | Van der Waals | Tetrameric | 0.6975 |  | |  | | |
| Arg186 sidechain | Glu494 sidechain | salt bridge | Dimeric | 0.69 | 2.137969986 **±**  0.761091751 | | 2.08075 **±**  0.671408 | | |
| Lys528 sidechain | Ala482 backbone | hydrogen bond | Dimeric | 0.655 | 1.753240738 **±**  0.091317639 | | 1.580459 **±**  0.177906 | | |
| Trp479 sidechain | Tyr532 sidechain | π-stacking | Tetrameric | 0.6325 | 10.34975783 **±**  2.062658885 | | 1.106752 **±**  0.867192 | | |
| Gly176 backbone | Asp177 backbone | Hydrogen bond | Tetrameric | 0.63 |  |  |  | |  |
| Val490 backbone | Val530 backbone | Hydrogen bond | Dimeric | 0.6125 | 0.08278286 **±**  0.148785021 | | 1.294107 **±**  0.396995 | | |
| Lys508 backbone | Glu526 sidechain | hydrogen bond | Dimeric | 0.61125 | 0.673170096 **±**  0.140826277 | | 0.298423 **±**  0.15761 | | |
| Pro182 sidechain | Glu501 sidechain | Van der Waals | Dimeric | 0.59 | 2.986942876 **±**  0.897385799 | | 2.610657 **±**  0.472427 | | |
| Trp166 sidechain | Tyr175 sidechain | hydrophobic contact | Tetrameric | 0.58 | 1.746332208 **±**  0.597700662 | | -0.58632 **±**  0.467321 | | |
| Ile179 sidechain | Glu501 backbone | Van der Waals | Dimeric | 0.575 | 2.46986626 **±**  0.873131742 | | 1.552609 **±**  0.109615 | | |
| Ile178 sidechain | Ile178 sidechain | hydrophobic contact | Tetrameric | 0.575 |  |  |  | |  |
| Gly533 Backbone | Asn493 | hydrogen bond | Dimeric | 0.5575 | -0.193829467 **±**  0.060566439 | | 5.472414 **±**  0.494131 | | |
| Val507 backbone | Glu526 sidechain | Van der Waals | Dimeric | 0.5525 | 1.746296615 **±**  0.115627526 | | 0.249582 **±**  0.469151 | | |
| Leu527 sidechain | Tyr519 sidechain | hydrophobic contact | Dimeric | 0.5325 | 8.703069296 **±**  0.285141807 | | 9.226687 **±**  0.772299 | | |
| Arg172 sidechain | Glu165 sidechain | salt bridge | Tetrameric | 0.53 | 5.574873412 **±**  0.567660245 | | -0.63661 **±**  0.432596 | | |
| Arg173 sidechain | Arg173 sidechain | Arginine π-stacking | Tetrameric | 0.53 |  |  |  | |  |
| Val300 sidechain | Gln 509 sidechain | Van der Waals | Dimeric | 0.5225 | 6.908860672 **±**  1.086210728 | | 1.8216 **±**  0.398842 | | |
| Leu293 backbone | Ala297 backbone | hydrophobic contact | Dimeric | 0.5125 |  | |  | |  |
| Tyr532 sidechain | Ile478 sidechain | hydrophobic contact | Dimeric | 0.5 | 4.047311935 **±**  1.115810974 | | 5.120799 **±**  0.463681 | | |
| Val190 sidechain | Ile178 sidechain | hydrophobic contact | Tetrameric | 0.5 | 5.062471207 ± 0.888366271 | | -0.282376182 **±** 0.159999289 | | |

**Table S2. Purification yield of hSSADH**

The purification steps (referred to as 1-liter culture) are described in the text. One unit of activity is defined as the amount of enzyme required to convert 1 μmol/min of NAD^+^ to NADH.

| Sample | Activity (U) | Proteins (mg) | Specific activity (U/mg) | Yield (%) | Purification (fold) |
| --- | --- | --- | --- | --- | --- |
| Crude extract | 787 | 1553 | 0.5 | 100 | 1 |
| Purified enzyme | 468 | 26 | 18.2 | 60 | 36 |

**Table S3. Pattern of NADH inhibition in the reaction catalyzed by hSSADH**

| Inhibitor pattern observed | | | | | | |
| --- | --- | --- | --- | --- | --- | --- |
| For varied SSA | | | | For varied NAD^+^ | | |
| At unsaturated NAD^+^ | | At saturated NAD^+^ | At unsaturated SSA | | | At saturated SSA |
| non-competitive | no inhibition | | competitive | | competitive | |

**Table S4. Tm values determined by CD thermal denaturation at 222 nm or by DSC**. Experiments were carried out in 100 mM potassium phosphate buffer at pH 8. Data are expressed as mean ± SEM.

|  | T_m_ CD at 222 nm | | | T_m_ by DSC |
| --- | --- | --- | --- | --- |
| hSSADH | | 53.1 ± 0.4 | 51 ± 1 | |
| hSSADH + 1 mM DTT | | 50.5 ± 0.1 | 50.1 ± 0.1 | |
| hSSADH + 0.2 mM NAD^+^ | | 56.9 ± 0.4 | 54.9 ± 0.2 | |

**Table S5.** **Predicted pKa values for catalytic and titratable active site residues not involved in binding.**

| Residue | propka | pyPKA | pKAI+ | DeepPKA | pKa-ANI | Average ± S. D. |
| --- | --- | --- | --- | --- | --- | --- |
| \| Cys340 \| \| --- \| | 13.07 | 10.35 | 10.88 | NA | NA | 11.43 ± 1.44 |
| Cys342 | 9.14 | 9.34 | 11.22 | NA | NA | 9.90 ± 1.15 |
| Glu306 | 7.61 | 5.21 | 5.74 | 5.32 | 5.62 | 5.61 ± 1.44 |
| Arg213 | 14.54 | N.A. | N.A. | N.A. | 14.54 | 14.54 |
| Lys214 | 9.66 | 14.1 | 13.2 | 10.79 | 11.56 | 11.55 ± 2.06 |
| Glu515 | 3.24 | 1.21 | 2.50 | 3.89 | 3.13 | 3.13 ± 1.15 |

N.A., not available

**Table S6. Values of the secondary structure thermal stability in the absence or presence of 100 mM NAD^+^ and equilibrium dissociation constant K_D_ of hSSADH variants for NAD^+^**. Experiments were performed in 100 mM potassium phosphate buffer, pH 8 by using 0.1 mM (monomer concentration for Lys214Ala, Glu306Gln, and Glu515Gln) or 0.4 mM (monomer concentration for Glu306Ala and Glu515Ala). Data are repeated in duplicate (CD) or triplicate (K_D_) and expressed as mean ± SEM.

| # | T_m_ (°C) at 222 nm  without NAD^+^ | T_m_ (°C) at 222 nm  with NAD^+^ | K_D_ (mM) |
| --- | --- | --- | --- |
| Lys214Ala | 53.1 ± 0.1 | 56.9 ± 0.1 | 2.5 ± 0.2 |
| Glu306Ala | 61.9 ± 0.2 | 63.6 ± 0.1 | 29 ± 6 |
| Glu306Gln | 64.5 ± 0.3 | 66.0 ± 0.7 | 4.3 ± 0.4 |
| Cys340Ala | 58 ± 1 | 58.9 ± 0.5 | -- |
| Cya340Ser | 61.5 ± 0.1 | 63.7 ± 0.5 | -- |
| Glu515Ala | 57.5 ± 0.2 | 58.0 ± 0.3 | 42 ± 24 |
| Glu515Gln | 54.0 ± 0.8 | 53.9 ± 0.1 | 19 ± 4 |

**Table S7** **Oligonucleotide sequences used for PCR-based site-directed mutagenesis of prokaryotic expression vectors.** All sequences are shown in 5´–3´ direction. F: forward, R: reverse.

| Variant | Primer | Sequence (5'-3') |
| --- | --- | --- |
| C340A | Fw | GTAACACCGGCCAAACCGCCGTTTGCAGCAACC |
|  | Rev | GGTTGCTGCAAACGGCGGTTTGGCCGGTGTTAC |
| C340S | Fw | CACCGGCCAAACCAGCGTTTGCAGCAACC |
|  | Rev | GGTTGCTGCAAACGCTGGTTTGGCCGGTG |
| E306A | Fw | CGTGTGAGCATGGCACTGGGTGGCCTG |
|  | Rev | CAGGCCACCCAGTGCCATGCTCACACG |
| E306Q | Fw | CGTGTGAGCATGCAACTGGGTGGCCTGG |
|  | Rev | CCAGGCCACCCAGTTGCATGCTCACACG |
| E515A | Fw | GCGGTCTGGGCCGTGAGGGCAGCAAGTATGG |
|  | Rev | CCATACTTGCTGCCCGCACGGCCCAGACCGC |
| E515Q | Fw | GCGGTCTGGGCCGTGAGGGCAGCAAGTATGG |
|  | Rev | CCATACTTGCTGCCCTCACGGCCCAGACCGC |
| K214A | Fw | GATGATCACCCGTGCAGTTGGTGCGGCGC |
|  | Rev | GCGCCGCACCAACTGCACGGGTGATCATC |

**SI References**

Awoonor-Williams E, Golosov AA, Hornak V. 2023. Benchmarking in silico tools for cysteine pk(a) prediction. J Chem Inf Model. 63(7):2170-2180.

Buslaev P, Aho N, Jansen A, Bauer P, Hess B, Groenhof G. 2022. Best practices in constant ph md simulations: Accuracy and sampling. J Chem Theory Comput. 18(10):6134-6147.

Cai Z, Luo F, Wang Y, Li E, Huang Y. 2021. Protein pk (a) prediction with machine learning. ACS Omega. 6(50):34823-34831.

Didiasova M, Cesaro S, Feldhoff S, Bettin I, Tiegel N, Fussgen V, Bertoldi M, Tikkanen R. 2024. Functional characterization of a spectrum of genetic variants in a family with succinic semialdehyde dehydrogenase deficiency. Int J Mol Sci. 25(10).

Gokcan H, Isayev O. 2022. Prediction of protein pk (a) with representation learning. Chem Sci. 13(8):2462-2474.

Henehan GT, Tipton KF. 1991. The effects of assay temperature on the complex kinetics of acetaldehyde oxidation by aldehyde dehydrogenase from human erythrocytes. Biochem Pharmacol. 42(5):979-984.

Kang JH, Park YB, Huh TL, Lee WH, Choi MS, Kwon OS. 2005. High-level expression and characterization of the recombinant enzyme, and tissue distribution of human succinic semialdehyde dehydrogenase. Protein Expr Purif. 44(1):16-22.

Kim YG, Lee S, Kwon OS, Park SY, Lee SJ, Park BJ, Kim KJ. 2009. Redox-switch modulation of human ssadh by dynamic catalytic loop. EMBO J. 28(7):959-968.

Micsonai A, Moussong E, Wien F, Boros E, Vadaszi H, Murvai N, Lee YH, Molnar T, Refregiers M, Goto Y et al. 2022. Bestsel: Webserver for secondary structure and fold prediction for protein cd spectroscopy. Nucleic Acids Res. 50(W1):W90-W98.

Munoz-Clares RA, Casanova-Figueroa K. 2019. The importance of assessing aldehyde substrate inhibition for the correct determination of kinetic parameters and mechanisms: The case of the aldh enzymes. Chem Biol Interact. 305:86-97.

Munoz-Clares RA, Diaz-Sanchez AG, Gonzalez-Segura L, Montiel C. 2010. Kinetic and structural features of betaine aldehyde dehydrogenases: Mechanistic and regulatory implications. Arch Biochem Biophys. 493(1):71-81.

Olsson MH, Sondergaard CR, Rostkowski M, Jensen JH. 2011. Propka3: Consistent treatment of internal and surface residues in empirical pka predictions. J Chem Theory Comput. 7(2):525-537.

Pometun AA, Parshin PD, Galanicheva NP, Shaposhnikov LA, Atroshenko DL, Pometun EV, Burmakin VV, Kleymenov SY, Savin SS, Tishkov VI. 2022. Effect of additional amino acid replacements on the properties of multi-point mutant bacterial formate dehyderogenase psefdh sm4s. Acta Naturae. 14(1):82-91.

Reis P, Bertolini M, Montanari F, Rocchia W, Machuqueiro M, Clevert DA. 2022. A fast and interpretable deep learning approach for accurate electrostatics-driven pk(a) predictions in proteins. J Chem Theory Comput. 18(8):5068-5078.

Reis P, Vila-Vicosa D, Rocchia W, Machuqueiro M. 2020. Pypka: A flexible python module for poisson-boltzmann-based pk(a) calculations. J Chem Inf Model. 60(10):4442-4448.

Valenzuela-Soto EM, Munoz-Clares RA. 1993. Betaine-aldehyde dehydrogenase from leaves of amaranthus hypochondriacus l. Exhibits an iso ordered bi bi steady state mechanism. J Biol Chem. 268(32):23818-23823.

Vojtechova M, Rodriguez-Sotres R, Valenzuela-Soto EM, Munoz-Clares RA. 1997. Substrate inhibition by betaine aldehyde of betaine aldehyde dehydrogenase from leaves of amaranthus hypochondriacus l. Biochim Biophys Acta. 1341(1):49-57.

Yammine A, Gao J, Kwan AH. 2019. Tryptophan fluorescence quenching assays for measuring protein-ligand binding affinities: Principles and a practical guide. Bio Protoc. 9(11):e3253.
